# Gel Electrolytes for Flexible Zinc‐Air Batteries: Design, Challenges, and Perspectives

**DOI:** 10.1002/advs.77061

**Published:** 2026-08-11

**Authors:** Yapeng Cao, Tengteng Gu, Yuqiong Wen, Xingxing Gao, Jian Wang, Jun Liu

**Affiliations:** ^1^ Science and Technology Center Affiliated Zhongshan Hospital of Dalian University Dalian Liaoning People's Republic of China; ^2^ College of Environment and Chemical Engineering Dalian University Dalian Liaoning People's Republic of China; ^3^ School of Materials Science and Engineering and Guangdong Provincial Key Laboratory of Advanced Energy Storage Materials South China University of Technology Guangzhou People's Republic of China

**Keywords:** dendrite suppression, flexible zinc‐air batteries, gel electrolytes, interface engineering

## Abstract

Flexible zinc‐air batteries (FZABs) have emerged as promising candidates for next‐generation flexible electronics and large‐scale energy storage. However, dendrite growth, interfacial side reactions, and electrolyte leakage severely limit their cycle life and energy density. This review systematically summarizes recent advances in advanced gel electrolytes for FZABs. We first establish a comprehensive classification framework and horizontal comparison of aqueous, ionic liquid, and organic gel electrolyte systems. The fundamental mechanisms of zinc dendrite growth, interfacial failure, and unique challenges under flexible conditions are thoroughly analyzed. Focusing on dendrite suppression and ultrathin design, we elaborate four core strategies: regulating ion transport for uniform deposition, enhancing mechanical/interfacial stability, optimizing solvation structures to suppress side reactions, and innovating in macroscopic architectures for functional integration. The inherent trade‐offs between ionic conductivity, mechanical robustness, and energy density are critically evaluated. Finally, future directions in multifunctional integration, advanced in‐situ characterization, and scalable manufacturing are discussed, providing theoretical guidance for developing high‐performance, safe, flexible energy storage devices.

## Graphical abstract

This review summarizes design strategies for gel electrolytes in flexible zinc‐air batteries, focusing on dendrite suppression, interfacial stability, and ultrathin architectures to enhance cycle life and energy density.

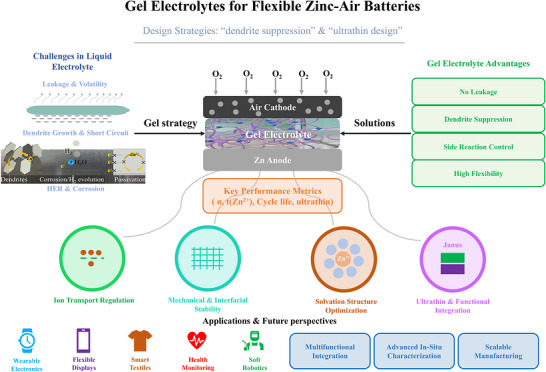

## 1 Introduction

The accelerating global transition toward a clean and low‐carbon energy system, coupled with the large‐scale integration of intermittent renewable sources such as wind and solar power, has created an urgent demand for efficient, low‐cost, and long‐duration large‐scale energy storage technologies. Concurrently, the rapid development of electric vehicles and portable electronics imposes increasingly stringent requirements on batteries regarding energy density, safety, cost, and sustainability [1, 2, 3]. Although lithium‐ion batteries (LIBs) currently dominate the markets for electric vehicles and smart devices, their further advancement faces multiple challenges. On one hand, key raw materials such as lithium, cobalt, and nickel are geographically concentrated [4]. Their mining and refining processes entail environmental and social risks, and supply chain vulnerabilities lead to significant price volatility, constraining long‐term sustainability and cost control [5, 6, 7]. On the other hand, the flammable organic electrolytes pose safety hazards, as they are prone to thermal runaway under conditions such as high temperature, overcharging, or physical damage [1, 4, 5]. Furthermore, the energy density of LIBs is approaching its theoretical limit, offering diminishing room for improvement. Their complex structural design and material composition also present significant hurdles for efficient and economical recycling [8, 9]. Therefore, the development of novel electrochemical systems with higher theoretical energy density and enhanced environmental friendliness is particularly imperative. Among the candidates, metal‐air batteries have recently garnered considerable research interest due to their inherent potential advantages.

Zinc‐air batteries (ZABs) stand as a compelling candidate for next‐generation energy storage, offering a combination of high theoretical energy density (∼1086 Wh·kg^−1^, approximately three times that of typical lithium‐ion batteries), low material cost, and favorable environmental compatibility [10, 11]. Their semi‐open structure, which directly utilizes atmospheric oxygen as the cathode active material, eliminates the need to store an oxidizer within the cell. This significantly reduces overall weight and volume, making ZABs particularly suitable for portable devices and wearable electronic systems [3]. The abundance of zinc in the Earth's crust (about 75 times that of lithium), coupled with its stable pricing and mature extraction processes, grants ZABs considerable economic viability and sustainability potential for large‐scale applications [12]. From a safety perspective, ZABs predominantly employ non‐flammable aqueous electrolytes, fundamentally avoiding the thermal runaway risks associated with organic electrolytes in LIBs. Furthermore, zinc itself exhibits low toxicity, and its recycling technology is relatively well‐developed, contributing to a reduced environmental footprint throughout its lifecycle [12, 13]. Additionally, with advances in flexible electronics, ZABs based on solid‐state or gel electrolytes demonstrate excellent mechanical adaptability. They can maintain stable power output under bending or stretching deformation, providing an ideal, lightweight, high‐energy power solution for emerging applications like smart textiles and health monitoring [14, 15, 16]. Despite these advantages, ZABs face a series of interconnected technical challenges in practical deployment [17]. On the zinc anode side, dendrite growth during cycling can lead to internal short circuits, while hydrogen evolution reactions and passivation phenomena reduce Coulombic efficiency and cause active material loss. The air cathode is limited by the sluggish kinetics of the oxygen reduction and evolution reactions, often requiring high‐cost catalysts [18, 19, 20]. It is also susceptible to carbonate formation from atmospheric CO_2_, which can block electrode pores [12, 21]. Regarding the electrolyte, water evaporation/absorption alters concentration, traditional liquid systems carry leakage risks, and solid electrolytes often suffer from high interfacial resistance and insufficient ionic conductivity [12, 13, 17]. These issues collectively result in a practical energy efficiency typically below 60%, with cycle life and mechanical flexibility also requiring significant improvement [19, 20, 22, 23]. Recent breakthroughs in electrolyte engineering have provided innovative paradigms for addressing these intrinsic limitations. For instance, Liu et al. [24] demonstrated a separated Zn‐air system (SZAS) that strategically decouples the charging process in near‐neutral electrolyte from discharging in alkaline electrolyte, achieving exceptional anode reversibility (99.84% Coulombic efficiency over 5000 cycles) and high discharge energy density (218 Wh·kg^−1^). While this system employs liquid electrolytes, its underlying dual‐electrolyte concept inspires the development of Janus‐type or asymmetric gel electrolytes capable of spatially segregating distinct electrochemical environments within a single quasi‐solid matrix, thereby simultaneously optimizing anode stability and cathode kinetics. Furthermore, innovative strategies for active dendrite management have emerged beyond passive suppression approaches. Current research is actively addressing these bottlenecks through strategies such as novel electrolyte design, interface engineering, and system optimization to advance their practical application.

In Flexible zinc‐air batteries (FZABs), gel electrolytes demonstrate significant potential to replace conventional liquid electrolytes, owing to their unique structural and performance advantages. The core mechanism lies in immobilizing the liquid components within a three‐dimensional polymer network, thereby creating a matrix that integrates the mechanical stability of a solid with efficient ionic conduction [25]. Compared to leak‐prone and volatile liquid systems, gel electrolytes offer superior flexibility and mechanical robustness. This enables them to accommodate structural changes in flexible devices during deformation while maintaining intimate electrode‐electrolyte interfacial contact. Consequently, they can effectively suppress uneven current distribution on the zinc anode surface, significantly mitigating zinc dendrite growth [23, 26, 27]. Furthermore, their high‐water retention capability and quasi‐solid characteristics help reduce water evaporation and electrolyte leakage. They also alleviate carbonate precipitation caused by the intrusion of atmospheric CO_2_, thereby enhancing the battery's cycle stability and safety [12, 28]. Research indicates that functional gel systems, such as composite electrolytes incorporating carboxymethyl cellulose or polyacrylamide, can further optimize zinc deposition behavior through the coordination between functional groups and Zn^2+^ ions, which enables stable cycling for over 1000 h [29, 30, 31]. Beyond passive suppression strategies, active dendrite management approaches have recently emerged as a complementary paradigm. A voltage‐controlled dendrite rearrangement (DR) strategy utilizing the reversible Br^−^/Br_2_ redox chemistry enables in‐situ elimination of pre‐formed zinc dendrites through temporary voltage elevation, which extends operational lifespans to 80 000 cycles in Zn‐ion hybrid capacitors and 3000 h in symmetric cells [32]. Although demonstrated in liquid electrolyte systems, this dynamic interfacial remediation principle underscores the importance of engineering gel electrolytes with responsive, self‐regulating interfacial chemistries that can actively adapt to evolving dendrite morphologies under the dynamic deformation conditions characteristic of flexible devices. The molecular‐level regulation of Zn^2+^ solvation structures has proven to be a particularly powerful strategy for suppressing parasitic reactions and stabilizing the zinc anode interface. For example, the introduction of p‐hydroxybenzaldehyde (M4) as an electrolyte additive reconstructs the [Zn(H_2_O)_6_]^2+^ primary solvation sheath and forms a protective adsorption layer on the Zn anode surface, effectively blocking proton corrosion and hydrogen evolution even in acidic electrolytes, thereby enabling Zn//MnO_2_ batteries to achieve over 3000 stable cycles with an average Coulombic efficiency exceeding 97.3% [33]. This additive‐mediated solvation engineering approach provides a direct molecular blueprint for the rational design of polymer matrices in gel electrolytes, wherein immobilized functional moieties‐such as carboxyl, hydroxyl, or aldehyde groups‐can similarly modulate the Zn^2+^ coordination environment, reduce free water activity, and construct adaptive electrode‐electrolyte interphases critical for long‐term cycling stability in flexible configurations. These characteristics establish gel electrolytes as a crucial materials platform for advancing ZABs toward flexibility, long cycle life, and enhanced safety.

**FIGURE 1:**
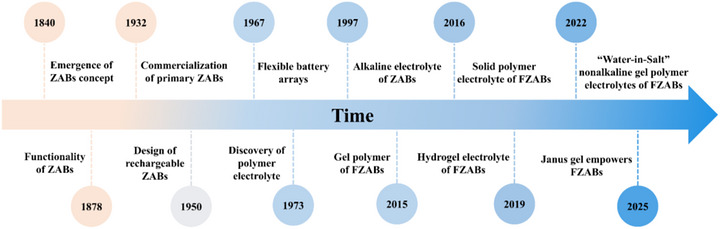
Timeline for the development of ZABs [34, 35, 36]. Adapted with permission [34]. Copyright 2024, Tsinghua University Press.

Figure 1 displays the timeline from ZABs to FZABs. As comprehensively summarized by Wu et al. [34], the conceptual foundation of ZABs was first proposed by Smee in 1840. Approximately thirty‑eight years later, Maiche successfully demonstrated the feasibility of primary ZABs using a porous platinum‑modified carbon material as the cathode. In 1932, Heise and Schumacher achieved the commercial‑scale production of ZABs with a neutral electrolyte system, marking a significant milestone in the field. Subsequently, the advent of polymer electrolyte materials provided a critical impetus for technological innovation in ZABs. During the 1970s, Wright and his research team introduced a composite electrolyte system based on poly (ethylene oxide) (PEO). Since 1997, ZABs employing alkaline electrolytes have gradually become the dominant direction in both academic research and engineering applications. Building on this foundation, gel polymer electrolytes, all‑solid‑state polymer electrolytes, and hydrogen‑related technologies have been successively incorporated into the electrolyte design and development of FZABs. In recent years, the “water‑in‑salt” system and the “Janus gel empowers” strategy have also been explored and applied to the electrolyte research of FZABs [35, 36].

The main application directions of materials for FZABs encompass wearable electronic devices, health monitoring and implantable devices, smart textiles, flexible displays and human‐machine interaction, flexible lighting and optoelectronic devices, soft robotics and actuators, as well as smart packaging and logistics, among others [34, 37, 38]. In the research of FZABs, the development of gel electrolytes must address not only the inherent limitations of traditional liquid electrolytes but also the unique challenges arising from the application scenarios of flexible devices. The design strategy of anchoring liquid components within a three‐dimensional polymer network enables gel electrolytes to achieve a dynamic balance between mechanical/structural stability and ion transport efficiency, while also providing innovative solutions for suppressing abnormal zinc dendrite growth and interfacial failure [22]. However, the dynamic deformation conditions faced by flexible devices during service demand greater environmental adaptability from the electrolyte material. This requires maintaining stable electrode‐electrolyte interfacial contact and consistent electrochemical performance under complex mechanical stresses such as stretching and bending. Recent studies have significantly enhanced the overall material properties by introducing functional nanofillers (e.g., SiO_2_ nanoparticles, cellulose nanofibrils, or graphene oxide nanosheets) to modify the electrolyte system [22, 29, 39]. Furthermore, by regulating the Zn^2+^ solvation sheath structure—such as through the coordination interaction between carboxymethyl cellulose and zinc ions—deep charge/discharge cycling stability exceeding 1000 h has been achieved [31, 40]. Furthermore, to achieve excellent mechanical adaptability of gel electrolytes, an ultrathin design is a key aspect. Although conventional thick‐film electrolytes (>200 µm) can provide stable ion transport, they significantly increase the volume and weight of the battery, limiting their application in wearable devices [41, 42]. Recent studies have successfully achieved the ultrathin construction of gel electrolyte membranes using precision fabrication techniques such as electrospinning, blade coating, and interfacial self‐assembly, reducing their thickness to below 50 µm [39, 43]. This ultrathin design significantly shortens the ion transport path at the microscopic level, effectively reducing the interfacial charge‐transfer resistance; at the macroscopic level, it endows the device with excellent flexibility and conformability to curved surfaces, enabling perfect adaptation to curved electrodes and the complex geometries of wearable devices [44]. However, the substantial reduction in thickness inevitably brings the dual challenges of decreased mechanical strength and increased risk of dendrite penetration. To address these issues, researchers have adopted innovative strategies such as constructing hierarchical pore structures, optimizing gradient crosslinking density, and performing surface functionalization modifications, thereby forming a three‐dimensional interconnected ion transport network within the ultrathin electrolyte membrane. Furthermore, by introducing a hybrid backbone structure that combines rigidity and flexibility, the structural integrity and dendrite‐penetration resistance of the ultrathin membrane are effectively enhanced, achieving synergistic optimization of ultrathin design and high performance [45, 46, 47]. These research breakthroughs provide crucial theoretical support and experimental foundations for the subsequent systematic exploration of dendrite suppression mechanisms and the design strategies for ultrathin electrolyte structures.

**FIGURE 2:**
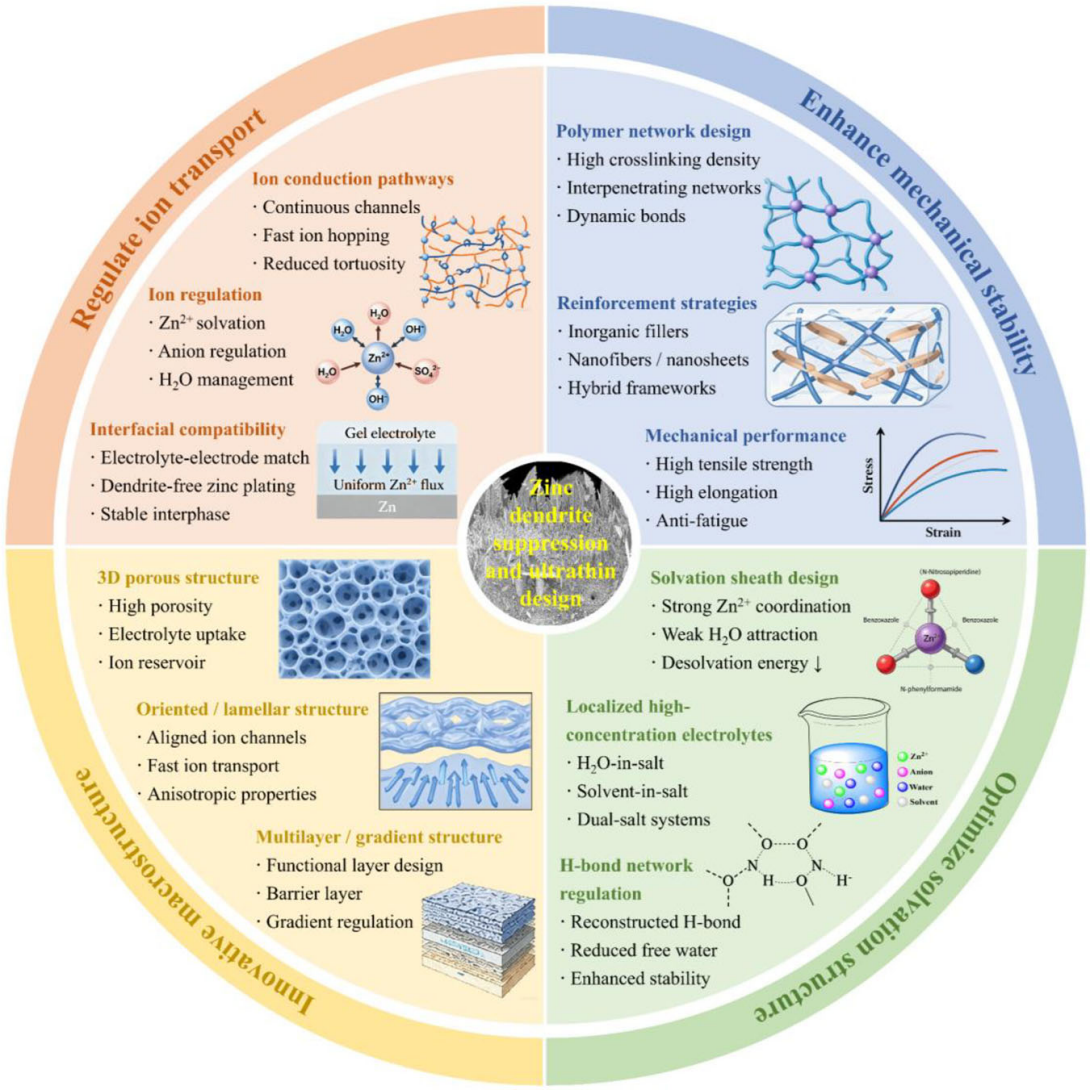
The review illustrates four dimensions of the design strategies and mechanisms of gel electrolytes.

The review aims to provide a systematic overview and critical assessment of recent advances in advanced gel polymer electrolytes for FZABs. We begin with an in‐depth analysis of the fundamental mechanisms underlying zinc dendrite growth and interfacial failure, highlighting the specific challenges these processes present under flexible conditions. Subsequently, focusing on the dual core objectives of dendrite suppression and ultrathin design, we elaborate on the design strategies and working mechanisms of gel electrolytes from four key perspectives: regulating ion transport, enhancing mechanical stability, optimizing solvation structures, and innovating macroscopic architectures (Figure 2). Based on this framework, a comprehensive evaluation of the key performance metrics of gel electrolytes, their corresponding battery‐level performance, and the associated challenges and trade‐offs is presented. Finally, we outline future perspectives for this field, emphasizing promising directions such as multifunctional integration, advanced characterization techniques, and scalable manufacturing processes. This discussion aims to provide scientific reference and theoretical guidance for developing next‐generation, high‐performance, and safe flexible energy storage devices.

## 2 Overview of Gel Electrolytes

Gel electrolytes, serving as quasi‐solid ionic conductors bridging the gap between liquid and solid electrolytes, confine liquid solvents (water or organic solvents) containing Zn^2+^ salts within a three‐dimensional polymer network, forming a unique system that combines the high ionic conductivity of liquid electrolytes with the mechanical stability of solid electrolytes [48, 49]. Their structural design relies on chemical crosslinking or physical entanglement, wherein hydrophilic groups within the polymer chains not only enhance structural integrity by increasing the crosslinking density but also modulate the Zn^2+^ solvation environment, effectively suppressing dendrite growth [50, 51]. In the context of flexible zinc‐air battery applications, gel electrolytes reduce the system's water activity through a “water‐locking” strategy, simultaneously suppressing hydrogen evolution reactions and metallic zinc corrosion. When combined with an ultrathin structural design, this approach significantly improves the energy density of the device. Furthermore, the incorporation of functional nanoadditives optimizes ion transport pathways, achieving a high Zn^2+^ transference number and thereby substantially enhancing the cycling stability of the battery [22, 43, 52]. This synergistic multi‐component mechanism provides an electrolyte solution that combines high safety with superior adaptability for flexible energy storage devices, demonstrating significant application potential.

### 2.1 Basic Composition and Classification

As a core functional material for FZABs, the comprehensive performance of gel electrolytes is essentially governed by the synergistic mechanism involving the three components: polymer matrix, solvent, and electrolyte salt. Through intermolecular interactions, these three components collectively establish ion transport pathways and a mechanical support network, directly determining key parameters such as ionic conductivity, mechanical strength, and electrochemical stability, thereby forming the physicochemical foundation for their functionality [53, 54, 55]. By systematically tailoring the structure of the polymer chains, the composition of the solvent system, and the type of electrolyte salt, precise optimization of the mechanical properties, ion migration behavior, and interfacial stability of gel electrolytes can be achieved, thus meeting the performance requirements of diverse application scenarios [30, 56, 57]. Based on differences in component characteristics, gel electrolytes can generally be classified according to the functional design of the polymer matrix, the choice of solvent system, and the type of electrolyte salt. Such a classification provides theoretical guidance for developing electrolyte systems tailored to specific needs [22, 58, 59].

While the above classification based on component characteristics provides a fundamental framework for understanding gel electrolytes, it is equally essential to compare these systems horizontally from the perspective of practical application requirements. In the context of FZABs, gel electrolytes can be broadly categorized into three representative systems according to their solvent types: aqueous gel electrolytes, ionic liquid gel electrolytes, and organic gel electrolytes. Each system exhibits distinctive physicochemical properties, electrochemical behaviors, and application scenarios, as systematically summarized in Table 1. This horizontal comparison enables rapid screening and rational selection of appropriate electrolyte materials for specific device configurations and operating conditions.

**TABLE 1:** Comparative overview of aqueous, ionic liquid, and organic gel electrolyte systems for flexible zinc‐air batteries.

System	Representative literature examples	Typical Solvent/Salt	Ionic conductivity	Electrochemical stability window	Mechanical flexibility	Primary advantages	Primary limitations	Optimal application scenarios	Ref.
Aqueous gel electrolytes	PAM‐SC, PAA‐PAM, CMC/PAM DN, PAGE, PAS‐CMC, MPS‐CNFs	H_2_O + ZnSO_4_, Zn(OTf)_2_, or KOH + Zn(Ac)_2_	10∼5×10^2^ mS·cm^−1^	Narrow (∼1.2–2.5 V)	Excellent; elongation at break 500%–2700%	High ionic conductivity comparable to liquid electrolytes; low cost; environmentally friendly; excellent water retention; facile fabrication; good biocompatibility.	Narrow electrochemical stability window due to high water activity; risk of water evaporation at elevated temperatures; susceptibility to CO_2_ absorption (carbonation); potential for hydrogen evolution at Zn anode.	Room‐temperature flexible ZABs; wearable electronics; health monitoring devices; large‐scale energy storage under mild conditions.	[29, 31, 60, 61, 62, 63, 64, 65]
Ionic liquid gel electrolytes	PAM‐PEGMA‐IL ionogel, IL‐modified PEO GPE, QSE‐P1I2 PPQAC membrane	EMIM‐TFSI, BMIM‐PF_6_ + Zn(TFSI)_2_	10^−1^–10^2^ mS·cm^−1^	Wide (>4 V)	Moderate; depends on polymer‐IL compatibility	Extremely wide electrochemical stability window; negligible vapor pressure (non‐volatile); non‐flammability; excellent thermal stability; suppressed CO_2_ absorption; suppressed Zn dendrite growth; extended cycle life.	Lower ionic conductivity than aqueous systems due to high viscosity; higher cost of ionic liquids; complex synthesis; potential toxicity of some ILs; limited ambient‐temperature performance without optimization.	High‐voltage flexible ZABs; wide‐temperature operation; aerospace and extreme environment applications; long‐cycle‐life energy storage.	[25, 44, 66, 67, 68, 69]
Organic gel electrolytes	PAB‐DMSO organohydrogel, DMSO/EC‐DEC GPE, glycerol‐modified PVA gel, GBL‐based GPE	DMSO, EC/DEC, GBL + Zn(TFSI)_2_, Zn(ClO_4_)_2_	10^−2^∼10 mS·cm^−1^	Moderate to wide (2–4 V)	Moderate to excellent; organic solvents plasticize polymer networks	Suppressed water activity via organic co‐solvent; broadened electrochemical stability window; excellent antifreeze properties; reduced hydrogen evolution; enhanced Zn^2+^ solvation sheath regulation; uniform Zn deposition.	Lower ionic conductivity than aqueous gels; higher cost of organic solvents; potential toxicity of some solvents; complex optimization of solvent/polymer compatibility; reduced water retention compared to aqueous systems.	Low‐temperature flexible ZABs; extreme climate wearable devices; high‐voltage Zn‐metal batteries; applications requiring wide operational temperature range.	[4, 43, 48, 70, 71, 72]

**FIGURE 3:**
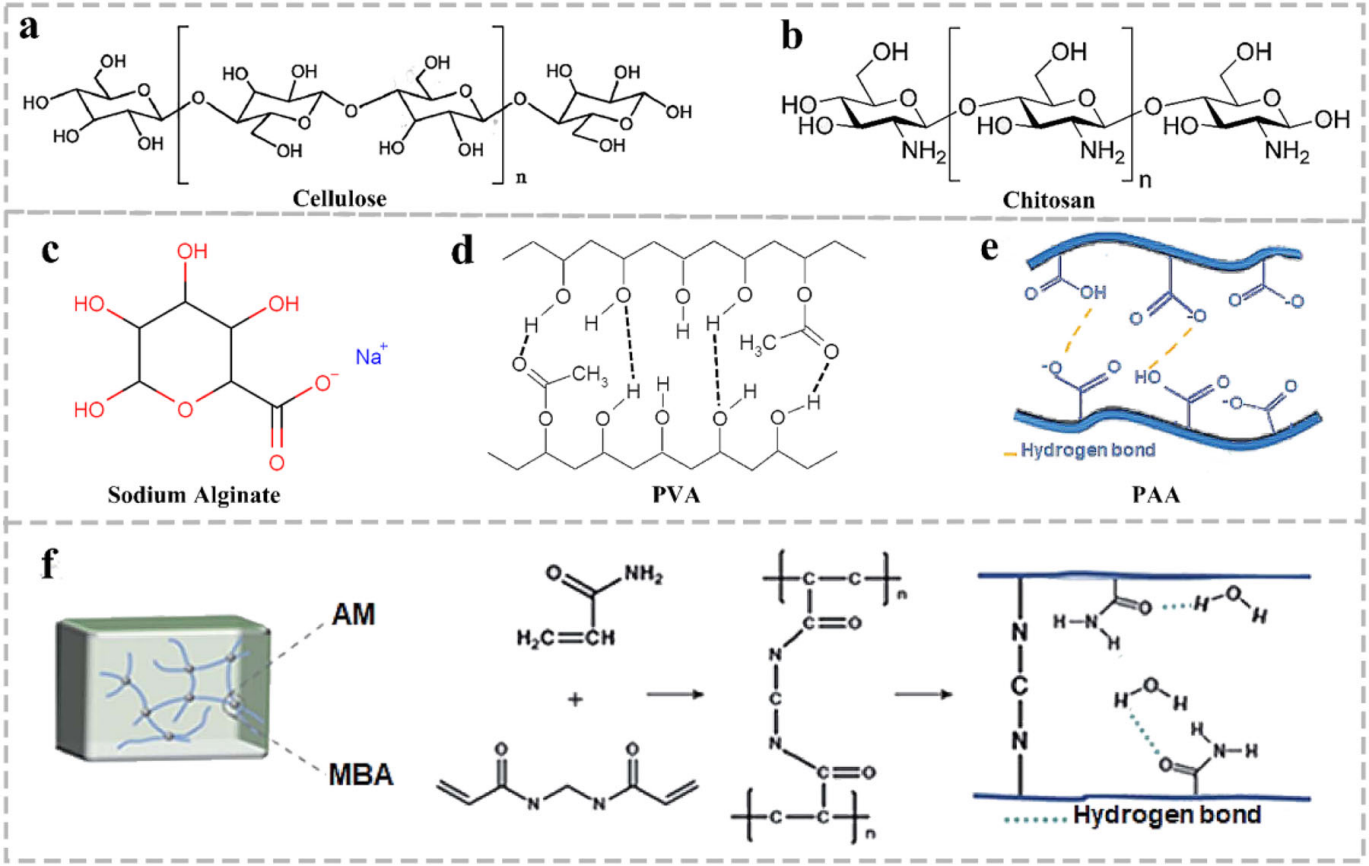
Natural polymer monomer structures: (a) Cellulose. (b) Chitosan. (c) Sodium alginate. Synthetic polymer structures: (d) PVA. (e) PAA. (f) Polymerization process of PAM. Reproduced with permission [48]. Copyright 2022, Royal Society of Chemistry.

The polymer matrix serves as the backbone of the three‐dimensional physical or chemical network of the gel, determining its macroscopic mechanical properties (e.g., modulus, toughness), thermal stability, and interfacial compatibility with electrodes [4, 57]. Based on differences in source, commonly used polymers can be classified into natural and synthetic categories: natural polymers (e.g., cellulose, chitosan, sodium alginate, with their structures shown in Figure 3a–c) have garnered attention due to their excellent biocompatibility and renewability, but their application is constrained by limitations such as insufficient molecular structural uniformity and limited tunability. In contrast, synthetic polymers (e.g., poly (vinyl alcohol) (PVA), poly (acrylic acid) (PAA), and polyacrylamide (PAM), with their structures shown in Figure 3d–f) dominate research owing to advantages such as controllable molecular weight distribution and high designability of functional groups [17, 20, 27, 73]. A common feature of these polymers is the presence of abundant strongly polar functional groups, such as hydroxyl (─OH), carboxyl (─COOH), and amide (─CONH_2_) groups, along the main chain [4]. These groups not only impart excellent liquid absorption and retention capabilities but also facilitate the construction of a three‐dimensional porous network through hydrogen‐bonding self‐assembly or chemical crosslinking (e.g., using crosslinkers such as glutaraldehyde or N, N’‐ methylene diacrylamide) [4, 20, 39]. This structure effectively immobilizes the liquid components to prevent leakage, inhibits zinc dendrite penetration through a physical barrier effect, and maintains the structural integrity of the electrolyte system [22, 34, 53, 74]. Notably, the systematic integration of diverse functional moieties—including sulfonate, amine, and cyano substituents—has been conclusively shown to enable precise modulation of the physicochemical characteristics of gel polymer electrolytes in alkali metal battery systems [4, 26, 75]. These findings offer critical insights for the rational design of FZABs by establishing structure‐property relationships that guide electrolyte optimization.

As the transport medium for ion migration, the physicochemical properties of the solvent play a decisive role in the ionic conductivity and electrochemical stability window of the electrolyte [76]. In flexible aqueous ZAB systems, water is the preferred solvent due to its superior ion dissociation capability and cost advantages; however, highly active free water molecules readily induce parasitic side reactions such as anodic hydrogen evolution and zinc corrosion, leading to performance degradation [13, 77]. To address this trade‐off, current research focuses on strategies for regulating water activity. On one hand, hydrogen‐bonding networks are constructed within the polymer matrix to convert free water molecules into bound water confined within the three‐dimensional network, thereby reducing their reactivity. On the other hand, organic co‐solvents such as ethylene glycol, glycerol, and dimethyl sulfoxide (DMSO) are introduced to achieve water dilution and activity suppression through reconstruction of the solvation structure. These synergistic approaches effectively broaden the electrochemical stability window of the electrolyte and inhibit side reactions [54, 57]. This dual‐pronged regulatory mechanism provides a critical solution for balancing the ionic conductivity and chemical stability of aqueous electrolytes.

As the primary source of charge carriers (predominantly Zn^2+^ and OH^−^) in gel electrolytes, the electrolyte salt, including its type, concentration, and anion characteristics, exerts a decisive influence on ion transport kinetics and the electrodeposition behavior of the zinc electrode [56, 59, 78]. In zinc‐based electrolyte systems, the anions of typical zinc salts such as Zinc sulfate (ZnSO_4_), Zinc trifluoromethanesulfonate (Zn(OTf)_2_), and Zinc chloride (ZnCl_2_) (i.e., SO_4_^2−^, OTf^−^, Cl^−^) participate in the construction of the primary solvation sheath of Zn^2+^, significantly altering its desolvation energy barrier and deposition overpotential characteristics. For example, “water‐in‐salt” or “water‐in‐gel” electrolyte systems based on high‐concentration Zn(OTf)_2_ can effectively reduce the content of free water molecules by reconstructing the Zn^2+^ solvation structure, thereby significantly suppressing hydrogen evolution and zinc corrosion while maintaining high ionic conductivity [79, 80, 81]. Notably, the concentration of the electrolyte salt directly modulates the Zn^2+^ transference number, enhancing this value, which optimizes the distribution of ion flux at the electrode surface and mitigates concentration polarization, thereby promoting uniform zinc electrodeposition through kinetic regulation and effectively inhibiting dendrite growth [82, 83, 84]. This multi‐dimensional synergistic regulation strategy provides a critical theoretical foundation for the development of high‐performance zinc‐based electrolytes.

In addition to the aforementioned conventional gel systems, several emerging gel electrolyte architectures have garnered significant attention in recent years, offering innovative solutions to the intrinsic trade‐offs between ionic conductivity, mechanical robustness, and interfacial stability. Dynamic covalent self‐healing gels, exemplified by PAA‐CNF‐Fe^3+^ hydrogels utilizing reversible metal‐ligand coordination bonds, enable autonomous repair of mechanical damage and restoration of interfacial integrity at the zinc anode, thereby extending battery cycle life under dynamic deformation conditions [66, 85]. Zwitterionic polymer gels, such as those incorporating poly (sulfobetaine methacrylate) (SBMA), leverage the “anti‐polyelectrolyte effect” to construct strong hydration layers around Zn^2+^ ions, effectively suppressing hydrogen evolution and dendrite growth through electrostatically neutral yet highly hydrophilic chain architectures [86]. Particularly noteworthy are deep eutectic solvent (DES)‐based eutectogels, which employ eutectic mixtures of hydrogen‐bond donors and acceptors (e.g., choline chloride/urea or ethylene glycol‐based systems) as the solvent phase instead of pure water or ionic liquids. These eutectogels exhibit exceptional thermal stability, non‐flammability, and a wide electrochemical stability window while maintaining adequate ionic conductivity, rendering them particularly suitable for wide‐temperature FZABs applications [87, 88]. The synergistic integration of these emerging design paradigms‐dynamic self‐healing chemistry, zwitterionic hydration regulation, and deep eutectic solvation‐provides a versatile materials platform for next‐generation gel electrolytes capable of operating under extreme mechanical and thermal conditions.

### 2.2 Key Characteristics and Advantages of Gel Electrolytes

As summarized in Table 1, aqueous gel electrolytes exhibit the highest ionic conductivity and lowest cost, making them the preferred choice for room‐temperature applications. However, their narrow electrochemical stability window and susceptibility to water evaporation limit their applicability under extreme conditions. In contrast, ionic liquid gel electrolytes offer an exceptionally wide electrochemical stability window and superior thermal stability, albeit at the expense of ionic conductivity and cost. Organic gel electrolytes, particularly those incorporating DMSO or glycerol as co‐solvents, strike a balance by suppressing water activity while maintaining adequate ionic conductivity, rendering them particularly suitable for low‐temperature and high‐voltage operations. Notably, the boundaries between these categories are becoming increasingly blurred with the emergence of hybrid systems, such as organohydrogels and water‐in‐salt gels, which synergistically combine the merits of multiple solvent types.

**FIGURE 4:**
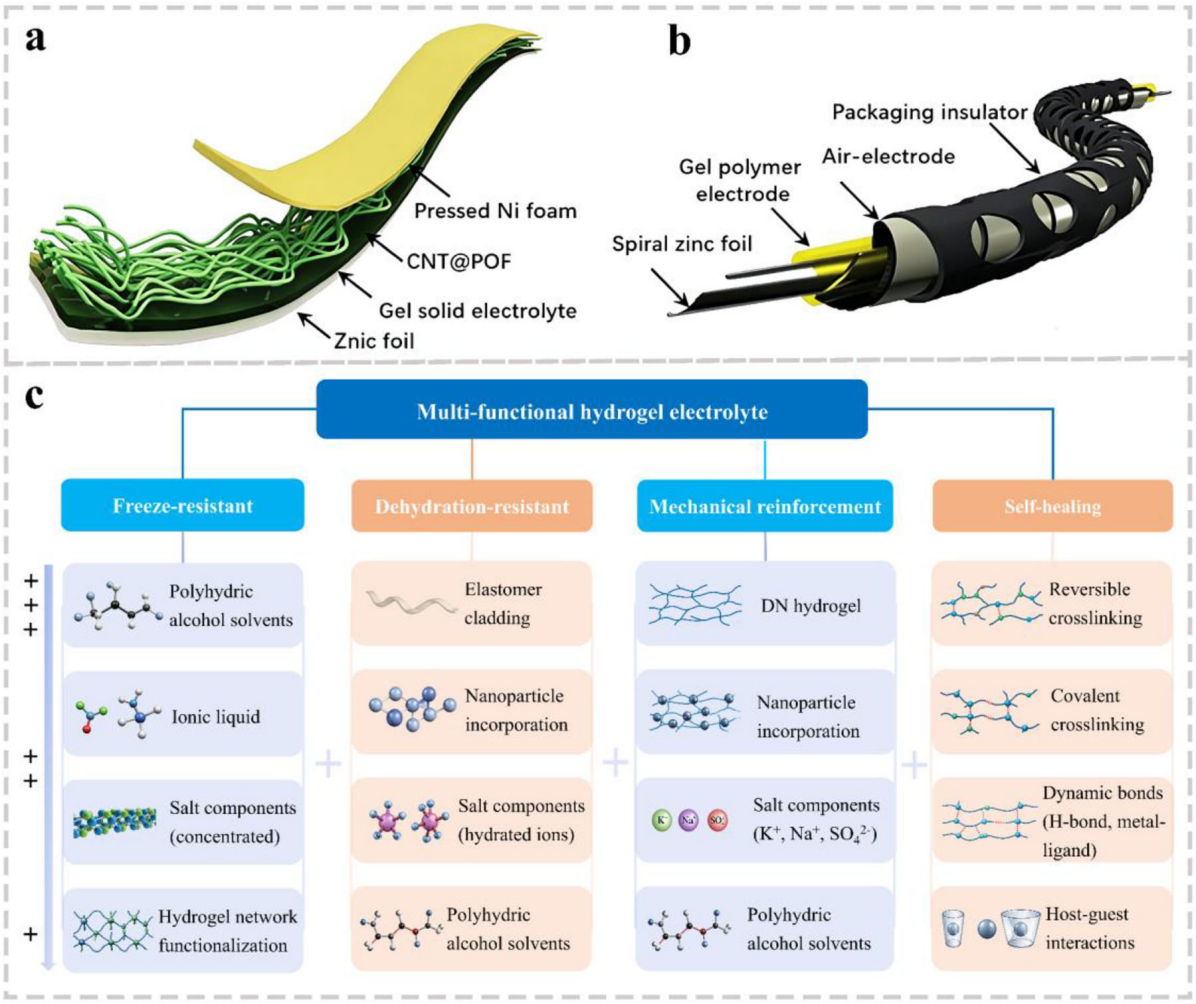
Schematic diagrams of FZABs with (a) sandwich. (b) Linear structure. Reproduced with permission [29]. Copyright 2024, Wiley‐VCH GmbH. (c) Synopsis of fabrication strategies for multi‐functional hydrogel electrolytes.

Compared with traditional liquid electrolyte systems, gel electrolytes exhibit significant synergistic advantages in FZABs applications. These advantages primarily stem from the physical confinement effects, chemical interactions, and mechanical adaptability induced by their quasi‐solid three‐dimensional network structure, as well as their tailorable chemical composition, offering innovative solutions to critical technical challenges in FZABs [34, 89]. Specifically, in terms of safety enhancement, the quasi‐solid characteristics of gel electrolytes are particularly prominent, as they completely eliminate the risk of leakage that conventional liquid electrolytes face when flexible devices are bent, compressed, or punctured [26, 55, 73, 75]. While traditional liquid electrolytes not only severely compromise battery performance upon bending or damage but also pose potential safety hazards, gel electrolytes firmly confine the liquid components within a three‐dimensional polymer network, maintaining structural integrity even under extreme mechanical deformation. This greatly improves the intrinsic safety of the battery and substantially expands its application potential in scenarios with stringent safety requirements, such as wearable electronic devices and flexible displays [34, 90]. Currently, in the structural application of gel electrolytes in FZABs, the device configurations mainly include sandwich‐type FZABs and fibrous FZABs (as illustrated in Figure 4a,b). Among these, the sandwich‐type FZABs consist primarily of a zinc electrode, a solid‐state electrolyte, and an air electrode. Due to its high structural similarity to conventional batteries, this configuration has been widely adopted in FZABs research and development [29]. In addition, gel electrolytes demonstrate comprehensive performance advantages in dendrite suppression, side reaction control, and mechanical flexibility, providing robust support for the further advancement of FZABs.

Gel electrolytes exhibit a dual mechanism in suppressing zinc dendrites. On one hand, their polymer network possesses a specific mechanical modulus (typically in the range of 0.1‐1 GPa, reaching MPa to GPa levels), enabling it to act as a physical barrier that directly impedes uncontrolled zinc dendrite growth and penetration, thereby effectively preventing internal short circuits in the battery. Experimental data indicate that gels with an elastic modulus exceeding the yield strength of zinc metal (approximately 0.8 GPa) can significantly enhance the cycle life of the battery. On the other hand, abundant hydrophilic or charged functional groups (e.g., ─COO^−^, ─SO_3_^−^) on the polymer chains engage in reversible electrostatic interactions with Zn^2+^, establishing highly selective Zn^2+^ transport pathways and substantially increasing the Zn^2+^ transference number [22, 34, 60, 91]. This effect not only guides the uniform distribution of Zn^2+^ during the initial stages of zinc deposition, homogenizing the ionic flux at the electrode surface, but also fundamentally suppresses dendrite nucleation and preferential growth driven by the “tip effect”—a capability that cannot be achieved by physical blocking alone. This dual mechanism provides a critical guarantee for the stable operation of FZABs.

In terms of side reaction control, gel electrolytes demonstrate exceptional performance, serving as another critical factor in enhancing battery cycle life [4, 92]. The hydrophilic groups within the polymer network, through dense hydrogen bonding interactions, convert a large number of water molecules from a highly active “free water” state to a low‐activity “bound water” state, achieving a unique “water state regulation” mechanism. This “water‐locking” effect greatly reduces the electrochemical activity of water, effectively suppressing hydrogen evolution and corrosion reactions at the zinc anode surface, thereby broadening the electrochemical stability window of the electrolyte [30, 34, 60]. Meanwhile, the incorporation of organic co‐solvents (e.g., DMSO, glycerol) further tunes water activity, while the adoption of “water‐in‐salt” electrolyte designs enables the reconstruction of the Zn^2+^ solvation sheath from the perspective of solvation structure, further reducing the amount of free water and creating a more stable interfacial environment for the zinc anode. Moreover, the introduction of organic co‐solvents also elevates the hydrogen evolution overpotential. Through the synergistic action of multiple mechanisms, the Coulombic efficiency of the zinc anode can be increased from approximately 80% in liquid systems to over 90%, significantly extending the cycle life of the battery [48, 83, 93, 94]. Figure 4c illustrates the design strategies for multifunctional hydrogel electrolytes. The various strategies listed in one column can be freely combined with those in the other columns to achieve a comprehensive construction of the hydrogel electrolyte. However, when performing such strategy combinations, several factors require careful consideration. The effects of strategies belonging to different columns vary significantly. Moreover, even the same strategy may exhibit different effects when applied to different functions of the hydrogel [72]. Table 2 summarizes representative gel electrolyte systems reported in recent literature, along with their key performance parameters.

**TABLE 2:** Some representative gel electrolyte systems.

Gel electrolyte system	Ionic conductivity (mS·cm^−1^)	Absorb electrolyte	Zinc dendrite suppression	Energy density / Specific capacity	FZAB cycle performance	Ref.
Alkaline PAA	—	6 M KOH + 0.2 M Zn(AC)_2_	Under low current density conditions, ZnO passivation layer forms on the surface of the zinc anode, leading to dendrite formation. Under high current density conditions, dendrites and by‐products are still observed on the zinc anode.	—	At 2 mA·cm^−2^, failure occurs after ∼133 h; at 10 mA·cm^−2^, after ∼50 h; and at 20 mA·cm^−2^, after ∼23 h.	[95]
HP‐PVA/PAA	212	6 M KOH	The semi‐interpenetrating network reduces local electric field concentration and inhibits protrusion nucleation.	—	45 cycles at 1 mA·cm^−2^	[45]
PAM‐SC	20°C: 324.68; −40°C: 73.96	6 M KOH	The polarized ─COO^−^ groups generate an electric field that homogenizes the Zn^2+^ flux; symmetric cells operate for 45 h without dendrites, as confirmed by COMSOL simulations demonstrating uniform deposition.	746.1 mAh·g^−1^	> 420 cycles at 0.5 mA·cm^−2^; 700 cycles at ‐40°C.	[60]
QSGPE	186	6 M KOH	The strong anionic ─SO_3_^−^ groups induce preferential deposition on the Zn (002) crystal plane, forming a dense layered structure.	242.23 mAh·cm^−3^	450 h of cycling at 1 mA·cm^−2^	[96]
WIS‐PAA/CNF	1.63	Zn(OTF)_2_	By means of strong hydrogen bonding and ion‐solvent interactions, the free water content is reduced, thereby suppressing hydrogen evolution and dendrite growth.	—	1300 h of cycling at 0.1 mA·cm^−2^	[35]
PMA‐HL	130.43	6 M KOH + 0.2 M Zn (CH_3_COO)_2_	La^3+^ preferentially adsorbs to form an electrostatic shielding layer; HACC restricts the diffusion of Zn(OH)_4_^2−^.	769 mAh·g^−1^	> 450 cycles at 1 mA·cm^−2^.	[97]
CPAM‐LC	327	6 M KOH	Rare earth ions preferentially adsorb on the (101) crystal plane of the zinc anode, inducing deposition on the Zn (002) crystal plane.	783 mAh·g^−1^	> 1350 cycles at 1 mA·cm^−2^.	[98]
PAS‐CMC	315.53	6 M KOH + 0.2 M Zn(AC)_2_	PAS‐CMC exhibits a strong interaction with the Zn (101) crystal plane, which is beneficial for interface stability.	808.87 mAh·g^−1^	> 120 h of cycling at 2 mA·cm^−2^.	[61]
mPAM	322	6 M KOH	—	—	110 h of cycling at 3 mA·cm^−2^.	[99]
PAA‐PAM	205	4 M KOH	The surface integrity of the PAA‐PAM gel after cycling is significantly higher than that of the single‐network gel, and its porous structure retains sufficient ion transport channels. Although a small number of dendrites are observed on the surface of the zinc anode.	788 mAh·g^−1^ at 2 mA·cm^−2^	97 h of cycling at 1 mA·cm^−2^.	[36]
PAA‐KC	165.4	7.5 M NaOH + 0.04 M ZnO	The polar groups of PAA and KC can modulate the solvation structure of Zn^2+^, reducing the coordination number of active water molecules; KC exhibits a lower adsorption energy on the Zn (002) surface, preferentially forming a protective film.	847.7 mAh·g^−1^	63 h of cycling at 2 mA·cm^−2^ (378 cycles).	[100]
ChCl/EG‐PVA	171.3	6 M KOH + 0.2 M Zn(Ac)_2_	Ch^+^ cations tend to preferentially adsorb onto the surface of the zinc anode, forming an electrostatic shielding layer and reconstructing the inner Helmholtz plane, thereby increasing the energy barrier for Zn^2+^ deposition and guiding uniform deposition.	782.5 mAh·g^−1^	More than 90 h of cycling at 2 mA·cm^−2^.	[87]
MPS‐CNFs‐KOH	318.21	6 M KOH + 0.2 M Zn(AC)_2_	The solvent‐rich phase provides uniform ion transport channels and homogenizes the Zn^2+^ flux; the polymer‐rich phase forms a physical barrier that effectively suppresses side reactions and dendrite growth.	726 mAh·g^−1^ at 2 mA·cm^−2^ and 732 mAh·g^−1^ at 5 mA·cm^−2^	> 107 h of cycling at 1 mA·cm^−2^.	[63]
PAGE	178	6 M KOH + 0.2 M Zn(AC)_2_	The ─COO^−^ groups homogenize the electric field and Zn^2+^ flux; the strong coordination between PDA and Zn^2+^ promotes desolvation of Zn^2+^ and suppresses water‐induced side reactions; a physical protection layer involving PDA is formed at the interface.	—	More than 40 h of cycling at 0.5 mA·cm^−2^.	[101]
M‐Agar	267	6 M KOH	By optimizing the interface, the formation of a relatively loose zinc oxide layer is promoted, which prevents the rapid formation of a dense insulating layer, thereby delaying failure caused by zinc passivation.	24 mAh·cm^−2^	25.1 h of cycling at 2 mA·cm^−2^ (75 cycles).	[102]
PAMNa‐CMCS	145.63	6 M KOH + 0.2 M Zn(AC)_2_	High adhesion ensures close contact between the electrolyte and the electrode, reduces interfacial impedance, homogenizes the ion flux, and suppresses dendrite growth.	745.82, 721.37, 704.34 mAh·g^−1^ at 1, 2, 5 mA·cm^−2^	86 h of cycling at 2 mA·cm^−2^.	[103]

In terms of mechanical flexibility, gel electrolytes exhibit exceptional mechanical‐electrochemical comprehensive performance, positioning them as critical materials in the field of flexible energy storage devices [22, 100]. The elasticity and extensibility imparted by the polymer network enable gel electrolytes to achieve an elongation at break of 500% ‐ 1000%, far exceeding that of conventional separator materials. This allows them to readily withstand repeated bending, folding, and even stretching deformations without structural failure, making them perfectly compatible with various complex mechanical deformation scenarios. Their soft nature offers a significant advantage by forming tight and conformal physical contact with electrodes, particularly with the rough surface of the zinc anode, thereby establishing a stable solid‐solid interface. This intimate contact not only substantially reduces interfacial resistance and ensures efficient ion transport but also adaptively maintains interfacial integrity during dynamic battery deformation, effectively preventing performance degradation caused by interfacial separation [34, 57, 100]. These comprehensive advantages position gel electrolytes as an indispensable “bridge” connecting high energy density, long cycle life, and excellent mechanical flexibility. They represent an important material platform for advancing next‐generation FZABs, laying a solid foundation for the large‐scale application of flexible batteries.

## 3 Fundamental Mechanisms of Zinc Dendrite Growth and Interface Failure

ZABs represent an advanced electrochemical energy storage technology characterized by high theoretical energy density, safety, environmental friendliness, and low cost, positioning them as a promising candidate for future flexible electronics and large‐scale energy storage. To advance their practical application and commercialization, a systematic improvement in their energy efficiency and cycling stability is essential. A critical bottleneck limiting battery cycle life and efficiency is the uncontrolled growth of zinc dendrites and the consequent interfacial failure. This phenomenon not only leads to capacity degradation but also poses safety risks such as internal short circuits. Therefore, a fundamental understanding of zinc deposition behavior, interfacial side reactions, and their specific failure mechanisms under flexible operation is crucial for developing zinc‐air batteries with long lifespan and high safety.

### 3.1 Zinc Deposition Kinetics and Dendrite Formation

**FIGURE 5:**
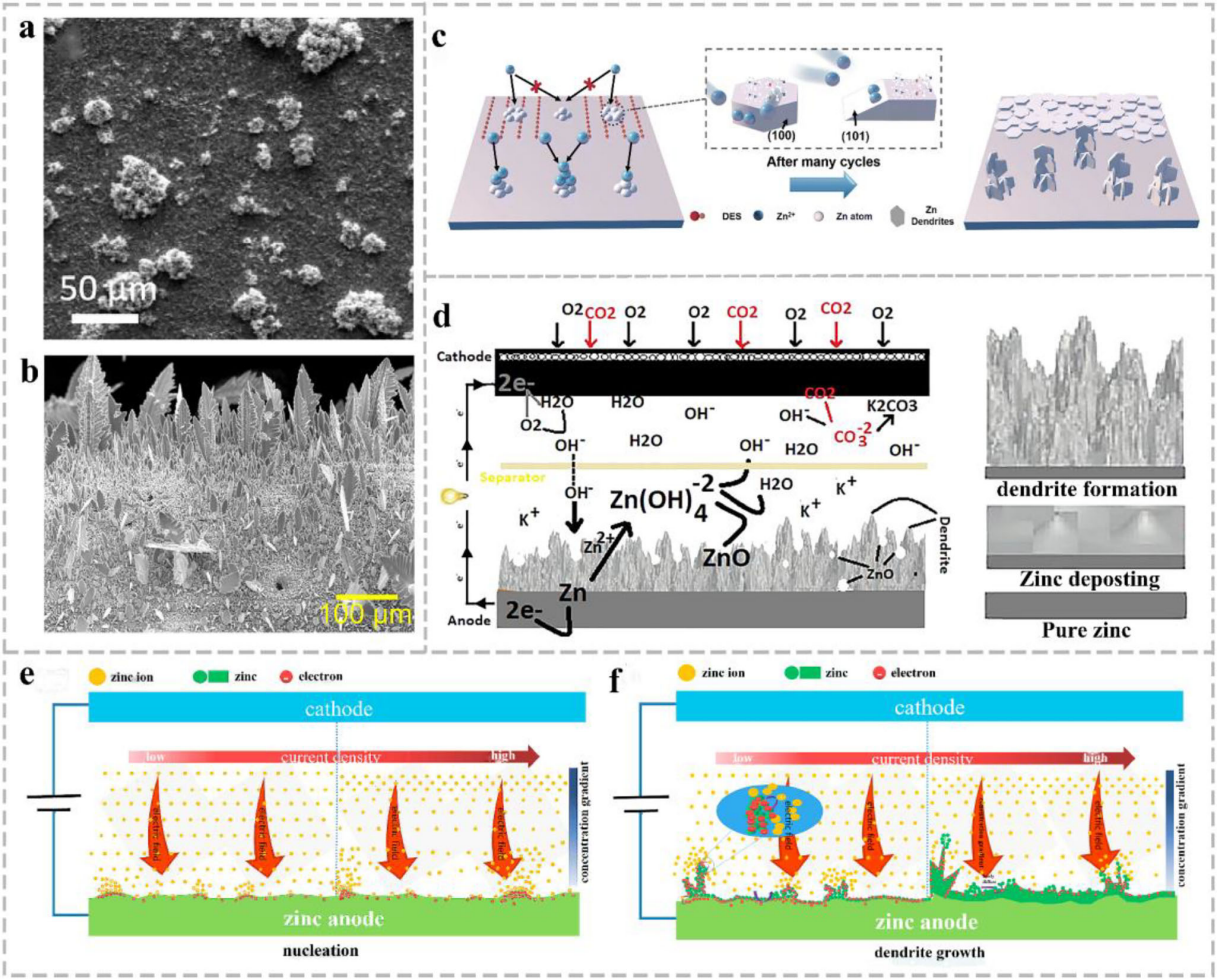
(a) Moss‐like zinc dendrites. Reproduced with permission [106]. Copyright 2024, American Chemical Society. (b) Dendritic zinc. Reproduced under the terms and conditions of the Creative Commons Attribution (CC BY) license [105]. Copyright 2024. Licensee MDPI, Basel, Switzerland. (c) Zinc Deposition Diagram. Reproduced under a Creative Commons Attribution 4.0 Unported license [41]. Copyright 2025, Royal Society of Chemistry. (d) Reactions in ZABs. Reproduced with permission [23]. Copyright 2020, Elsevier B.V. All rights reserved. Zinc dendrite formation stage: (e) Nucleation stage and (f) dendrite formation stage. Reproduced with permission [110]. Copyright 2021, Elsevier B.V. All rights reserved.

During the charge/discharge cycles of liquid ZABs, the metallic zinc anode undergoes the reversible deposition and dissolution reaction (Zn^2+^ + 2e^−^ ⇌ Zn) [4, 104]. An ideal deposition process should yield a flat and compact zinc layer. However, in practical alkaline electrolytes, zinc deposition often manifests in mossy or dendritic morphologies due to the combined effects of localized electric field enhancement and limited ion transport (Figure 5a,b) [105, 106, 107].

The deposition behavior of the zinc anode is governed by the electric field distribution, which is strongly modulated by surface micro‐geometry. Surface imperfections or high‐curvature protrusions concentrate the electric field (the “tip effect”, Figure 5c) [23, 41, 108], leading to higher local charge density and deposition current density at tips than on flat regions. This inhomogeneity drives preferential Zn^2+^ reduction at tips, establishing positive feedback that promotes rapid vertical growth and dendrite formation (Figure 5d) [23, 29, 109]. The deposition kinetics exhibit distinct stage‑dependent characteristics, which can be broadly categorized into nucleation and growth phases (Figure 5e,f). The nucleation stage requires overcoming a relatively high interfacial energy barrier (approximately 86–396 mV), whereas the subsequent growth stage involves a significantly lower barrier (42–220 mV) [110]. Under high current density, ions deposit preferentially at existing active sites rather than forming new nuclei, accelerating vertical dendrite propagation [109]. Notably, the deposition morphology is also closely related to the applied overpotential. At low overpotentials, zinc ions diffuse along the sides of dendrites toward the tips, resulting in a tip‑growth mode that forms fractal structures [31]. Morphology also depends on overpotential: at low overpotentials, ions diffuse along dendrite sides to tips, yielding fractal tip‐growth; at high overpotentials, direct root deposition produces needle‐like vertical structures [79, 111, 112]. Meanwhile, limited diffusion of zincate ions (Zn(OH)_4_^2−^) from the bulk causes interfacial depletion and concentration polarization at high currents. When polarization intensifies, the process shifts from kinetic control to mass‐transfer control [23, 60], which favors continued deposition at regions with higher local ion concentration and promotes three‐dimensional growth that aggravates dendrite branching and structural complexity [113].

Mechanical deformation‐bending, folding, and stretching‐poses critical challenges to gel electrolytes in FZABs by compromising both interfacial contact and electrochemical stability. Under deformation, the physical contact between the zinc anode and the gel electrolyte weakens, disrupting ion transport pathways and creating localized current density gradients that accelerate dendrite nucleation and growth [114]. Tensile stress further exacerbates these issues by increasing the porosity of the gel electrolyte, which disturbs the uniform flux of Zn^2+^ ions and promotes heterogeneous zinc deposition. The resulting dendrites, which possess a high Young's modulus (∼108 GPa), can readily penetrate the electrolyte layer and trigger internal short circuits during repeated cycling [62]. Concurrently, dynamic deformation undermines the chemical stability of the polymer matrix. Polymer chain cleavage induced by tensile stress can lead to electrolyte leakage and accelerated zinc anode corrosion [114]. The synergistic deterioration of interfacial and chemical stability significantly shortens the battery's cycle life. Studies indicate that gel electrolytes with enhanced mechanical properties and optimized anode–electrolyte interfaces are critical for suppressing dendrite formation and maintaining electrochemical performance under deformation. For instance, double‐network hydrogel electrolytes and deep eutectic solvent‐based gel polymer electrolytes have been demonstrated to improve tensile strength, electrolyte retention, and dendrite suppression, thereby enabling stable operation under bending conditions [88]. In a word, the synergistic effect of mechanical stress and electrochemical degradation leads to rapid capacity fading and eventual battery failure.

To address the challenges of dendrite growth and concentration polarization, various mitigation strategies have been proposed and experimentally validated. Three‐dimensional porous electrode designs, such as those incorporating carbon fiber scaffolds or porous zinc foil structures, effectively disperse current density and reduce local electric field intensity [115, 116]. For instance, a zinc symmetric cell employing a polyaniline (PANI)‐modified separator (GF/PANI‐600) demonstrated stable cycling for 3000 h at a current density of 1 mA·cm^−2^, whereas a cell with an unmodified separator (GF) failed after only 70 h. This confirms the significant effect of uniform electric field distribution on dendrite suppression [117]. Artificial interphase layer technology involves coating the zinc anode surface with composite layers (e.g., ZnF_2_/Zn_3_(PO_4_)_2_) to regulate the interfacial electric field. XPS analysis indicates that such interphases can lower the nucleation overpotential and guide uniform zinc ion deposition [117, 118]. Furthermore, double‐network hydrogel electrolytes (e.g., polyacrylic acid‐polyacrylamide, PAA‐PAM) can buffer mechanical stress through elastic modulus matching (1–10 MPa), maintaining intimate electrode/electrolyte contact and effectively reducing local electric field distortion [30, 116, 119]. Electrolyte optimization strategies, including the use of high‐concentration electrolytes or additives like citrate buffers, enhance the Zn^2+^ reservoir capacity [120, 121]. Mass transport enhancement strategies, exemplified by gel electrolytes with gradient pore structures or aligned ion channels using nanofibrillated cellulose, promote directional Zn^2+^ transport [122]. Interface engineering strategies construct zinc‐affinity layers (e.g., polyaniline‐modified surfaces) on the electrode, which chemically adsorb Zn^2+^ to lower the nucleation barrier and achieve uniform nucleation [80, 116, 117, 123]. In summary, for the design of gel polymer electrolytes in FZABs, strategies focusing on modulating ion transport, enhancing mechanical stability, and optimizing interfacial properties have shown significant effectiveness in suppressing zinc dendrite growth and alleviating concentration polarization. Future research needs to further balance the mechanical and electrochemical performance of these electrolytes to advance their practical application in flexible energy storage devices.

### 3.2 Interface Side Effect Impact

**FIGURE 6:**
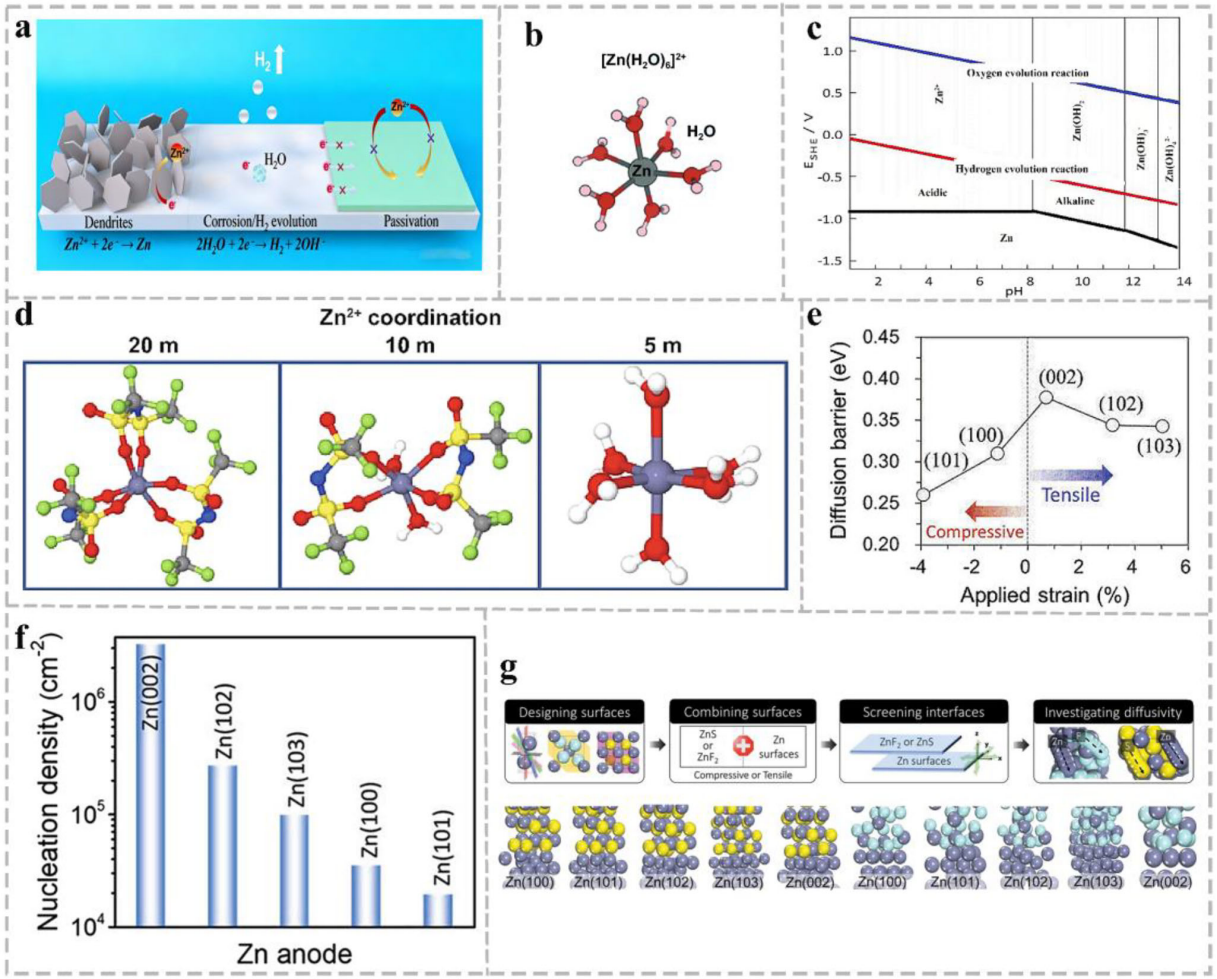
(a) Side reactions of zinc anodes in aqueous electrolytes (hydrogen evolution, corrosion, and passivation mechanisms). Reproduced with permission [128]. Copyright 2021, Elsevier B.V. All rights reserved. (b) Coordination environment of Zn^2+^ in H_2_O. Reproduced under the terms of the Creative Commons CC BY license [80]. Copyright 2022, Springer Nature. (c) Pourbaix diagram of zinc at different pH values. Reproduced with permission [23]. Copyright 2020, Elsevier B.V. All rights reserved. (d) Representative Zn^2+^ solvation structures in the electrolytes with 1 M Zn(TFSI)_2_ and three concentrations of LiTFSI (5, 10, and 20 M). Reproduced under the terms of the Creative Commons CC BY license [80]. Copyright 2022. Springer Nature. (e) Relationship between diffusion barriers and strain on different zinc crystal facets. (f) Differences in nucleation density of different Zn crystal planes. (g) Theoretical demonstration of design workflow for rational heterojunction structures between Zn and ZnS (111) and/or ZnF_2_ (111) with preferred orientations of Zn (Top row). Yellow and blue balls denote the sulfur and fluorine atoms, respectively. ZnF_2_ (111) and ZnS(111) surfaces can minimize the lattice mismatching to form the most stable heterojunctions with chemical bonds of Zn‐F and Zn‐S by five different Zn planes ((100), (101), (102), (103), (002)), based on the experimental XRD result in g). (Bottom row). Reproduced under the terms of the Creative Commons CC BY license [129]. Copyright 2023, Advanced Materials published Wiley‐VCH GmbH.

Zinc anodes in liquid ZABs suffer from significant hydrogen evolution reaction (HER) and corrosion issues across alkaline, neutral, and acidic electrolyte systems (Figure 6a) [108]. Thermodynamically, the standard redox potential of the Zn^2+^/Zn couple (‐0.76 V vs. SHE) is considerably more negative than the potential for water reduction (0 V vs. SHE). This inherent characteristic predisposes zinc to spontaneous reaction with water across all pH ranges (Figure 6b) [23, 80, 124]. Specifically, in alkaline electrolytes (e.g., KOH solution), zinc reacts with hydroxide ions to form soluble zincate ions (Zn(OH)_4_^2−^). Upon reaching supersaturation, these ions precipitate as zinc oxide (ZnO), a process accompanied by hydrogen gas evolution (Equations (1), (2), (3)) [12, 29]. The Pourbaix diagram in Figure 6c quantifies the stable phases and reaction pathways of zinc at different pH levels, confirming the tendency for Zn(OH)_4_^2−^ and ZnO formation under alkaline conditions [23]. In neutral electrolytes (e.g., ZnSO_4_ solution), zinc ions can form detrimental by‐products like [Zn_4_(OH)_6_SO_4_·*x*H_2_O] through complexation with sulfate and hydroxide ions (Equation 4). This side reaction leads to irreversible loss of active zinc and a significant increase in electrode interfacial impedance [29, 80]. In acidic environments, protons are directly reduced to hydrogen gas (Equation 5), accelerating zinc anode corrosion and posing safety risks such as cell swelling [23, 29]. The kinetics of HER are governed by electrolyte pH, local concentration gradients, and electrode polarization effects. Although the high overpotential for HER on zinc surfaces inherently limits its rate, this overpotential can be substantially reduced under high current densities or upon the formation of a ZnO passivation layer, thereby accelerating HER [23, 29, 125]. It is noteworthy that the continuous accumulation of hydrogen gas generated by HER increases internal pressure, potentially leading to electrolyte leakage and structural failure. A synergistic interplay exists between corrosion and HER: the formation of porous zinc deposits increases the electrochemically active surface area, altering local current density distribution and creating more favorable conditions for HER [23, 126, 127]. Concurrently, the hydroxide ions produced by HER alter the local chemical environment, accelerating zinc corrosion and establishing a detrimental corrosion‐HER feedback loop [23, 29]. As an illustrative example, Yang et al. [80] utilized molecular dynamics (MD) simulations to study an ultrahigh‐concentration electrolyte system consisting of 1 M Zn(TFSI)_2_ and 20 M LiTFSI. In this system, the Zn^2+^ solvation structure is fundamentally altered: Zn^2+^ ions are coordinated exclusively by TFSI^−^ anions, while water molecules are effectively screened by TFSI^−^ (Figure 6d). This electrolyte design drastically reduces water activity and prevents direct contact between water and the anode interface, thereby suppressing the desolvation process and significantly inhibiting both HER and anode corrosion.

Zinc dissolution and hydrogen evolution in alkaline electrolyte:

(1) $$Zn+4OH^{-}\to Zn{\left(OH\right)}_{4}^{2-}+2e^{-}$$

(2) $$Zn{\left(OH\right)}_{4}^{2-}\to ZnO+H_{2}O+2OH^{-}$$

(3) $$2H_{2}O+2e^{-}\to H_{2}↑+2OH^{-}$$

By‐product formation in neutral electrolytes:

(4) $$4Zn^{2+}+6OH^{-}+SO_{4}^{2-}+xH_{2}O\to Zn_{4}{\left(OH\right)}_{6}SO_{4}\cdot xH_{2}O$$

Hydrogen evolution in acidic electrolytes:

(5) $$2H^{+}+2e^{-}\to H_{2}↑$$

The solid electrolyte interphase (SEI) formed on the zinc anode surface primarily consists of electrolyte decomposition products, including ZnO, Zn(OH)_2_, ZnCO_3_, and ZnF_2_. The structural stability of this SEI is critical to the overall battery performance [30, 79]. In principle, an effective SEI requires three key properties: high ionic conductivity (>10^−3^ S·cm^−1^), excellent electronic insulation (electronic conductivity <10^−10^ S·cm^−1^), and sufficient mechanical strength (elastic modulus >1 GPa) [79, 130]. However, in practice, SEI failure often occurs through several mechanisms. First, chemical dissolution of components like Zn(OH)_2_ and ZnCO_3_ can take place in alkaline electrolytes. Second, mechanical stress arising from the periodic volume changes of the zinc electrode during deposition/dissolution cycles can cause SEI fracture. Third, compositional inhomogeneity is common; traditional SEI often exhibits a mixed inorganic‐organic structure where varying ionic conductivities among different components lead to localized current focusing [30, 131, 132]. To construct stable and efficient SEI, recent research has focused on regulating its composition and structure via interface engineering. For instance, Shinde et al. [129] employed density functional theory (DFT) calculations to elucidate the fundamental mechanism of elastic SEI formation assisted by fluorine/sulfur‐based species, the diffusion kinetics enabling high Coulombic efficiency (CE), and its ideal effect on suppressing zinc dendrites (Figure 6e,g). The DFT results revealed that the Zn (101) crystallographic plane possesses the lowest surface diffusion barrier compared to the pristine surface and other planes such as (002), (102), and (103). It also exhibits higher surface energy at sulfur, fluorine, and zinc sites while enduring minimal compressive strain. These characteristics collectively promote the spontaneous epitaxial reorientation of zinc grains toward the electrode surface, facilitating the formation of a dense and stable SEI layer. Furthermore, the Zn (101) plane shows the lowest in‐plane nucleation density among all orientations (Figure 6f). As the density of nucleation sites increases, adjacent nuclei compete for the limited supply of zinc ions, causing two‐dimensional lateral diffusion to become dominant and consequently slowing the crystal growth rate.

The aforementioned theoretical calculations provide a mechanistic understanding of the performance variations in SEI layers formed on different crystal planes. Experimental studies further confirm that the Zn (101) plane facilitates the formation of an ultra‐thin (∼10.2 nm) SEI film enriched with ZnF_2_ and ZnS. This compact layer effectively suppresses zinc dendrite growth and parasitic reactions due to its high interfacial energy and extremely low electronic conductivity [79, 129]. In contrast, the SEI layer formed on the Zn (002) plane exhibits significant defects. It is notably thicker (>50 nm), structurally loose, and contains zinc carbonate species, all of which contribute to inferior battery performance [129, 133]. When the SEI layer lacks sufficient stability, it triggers a cascade of detrimental effects. Zinc deposits can become electrically isolated from the current collector due to SEI fracture, forming “dead zinc”. Concurrent parasitic reactions continuously deplete Zn^2+^ ions and solvent molecules from the electrolyte. Furthermore, localized current focusing can induce localized overheating, posing a potential thermal runaway risk [30, 79, 125, 134].

In response to the aforementioned challenges, current research primarily explores three strategic approaches. The first strategy focuses on electrolyte optimization, such as introducing fluoroethylene carbonate (FEC) into the electrolyte. This additive promotes the formation of a dense SEI layer rich in ZnF_2_. The ionic conductivity of this optimized SEI is reported to be up to two orders of magnitude higher than that of conventional SEI layers, significantly enhancing ion transport efficiency [79, 125, 133]. The second strategy involves the artificial construction of an SEI. For example, zinc alginate hydrogels can form a dynamic SEI layer via in situ cross‐linking reactions. This dynamic layer can adapt to the volume changes of the electrode material during cycling and possesses the ability to self‐heal microcracks, thereby effectively maintaining interfacial stability [30]. The third strategy centers on crystallographic plane regulation. Studies indicate that preferential exposure of the Zn (101) plane reduces the diffusion barrier for Zn^2+^ ions compared to the Zn (002) plane. This reduction facilitates more uniform zinc deposition, subsequently improving the cycling stability and overall performance of the battery [129].

### 3.3 Special Challenges Under Flexible Conditions

In the practical application of FZABs, gel electrolytes face dual challenges of interfacial and electrochemical stability when subjected to mechanical deformation, such as bending, folding, or stretching [22]. Research has revealed that dynamic stress can directly disrupt the physical contact at the electrode‐electrolyte interface, leading to obstructed ion transport pathways and uneven distribution of local current density [22, 29, 90, 130]. For instance, when a battery is bent to 90°, the contact area between the zinc anode and the gel electrolyte can decrease by approximately 30%. This reduction causes localized enrichment of Zn^2+^ ions, thereby accelerating the nucleation process of dendrites [22, 50]. Furthermore, tensile stress exacerbates these issues. Under 100% strain, the increased porosity of the electrolyte disturbs Zn^2+^ flux. This promotes the preferential growth of dendrites along the (002) crystallographic plane, which itself becomes more exposed under mechanical stress [40]. Experimental data indicate that after 500 stretch‐release cycles, the probability of dendrite penetration through the electrolyte dramatically increases from an initial 5% to 42%, directly leading to internal short circuits [52, 135].

Dynamic deformation not only compromises interfacial stability but also undermines the chemical stability of the gel electrolyte. Under bending conditions, the diffusion rate of OH^−^ ions within the electrolyte can decrease by up to 50%. The sluggish transport retards the oxygen reduction reaction (ORR) kinetics at the cathode, leading to an increase in cell polarization voltage of approximately 80 mV [18, 22, 30, 136]. Concurrently, tensile stress can induce cleavage of polymer chains, causing leakage of the KOH electrolyte. This leakage triples the corrosion rate of the zinc anode [137]. The synergistic deterioration of both interfacial and chemical stability significantly shortens the battery's cycle life. For instance, under 300% strain, the capacity retention of FZABs plummets from an initial 98.7% (at 0% strain) to 54.1% after only 200 cycles [28, 138]. Furthermore, folding operations can generate microcracks at the interface, which act as preferred channels for zinc dendrite initiation and growth. Three‐dimensional imaging via time‐of‐flight secondary ion mass spectrometry (ToF‐SIMS) reveals that the surface density of zinc dendrites on a folded electrode is six times higher than on a flat one. Moreover, these dendrites exhibit fractal morphologies that facilitate deeper penetration into the electrolyte layer [110, 134].

## 4 Performance Evaluation and Challenge Analysis

Based on a thorough understanding of zinc deposition kinetics and dendrite formation, interfacial side reaction mechanisms, and the distinctive failure modes under flexible conditions, it is essential to establish a direct connection between these fundamental insights and the subsequent strategies for gel electrolyte design as well as the associated performance evaluation criteria. In particular, the tip effect and concentration polarization during dendrite formation impose rigorous demands on ionic conductivity and Zn^2+^ transference number‐only when ion transport is sufficiently rapid and the Zn^2+^ flux distribution is spatially uniform can the incipient nucleation of dendrites be effectively suppressed [139, 140]. Concurrently, parasitic reactions such as hydrogen evolution and zinc corrosion are governed primarily by the electrochemical stability window and the water‑retention capability of the gel electrolyte; hence, widening the stability window and reducing water activity through solvation structure regulation and polymer network architecture are required [141, 142]. Moreover, the mechanical modulus and interfacial compatibility collectively determine the physical barrier efficacy of the gel electrolyte against dendrite penetration and its adaptability to volume fluctuations [143]. Consequently, a comprehensive assessment of these parameters offers critical guidance for the rational formulation of gel electrolytes, with the ultimate aims of inhibiting dendrite proliferation and securing long‑term cycling durability.

### 4.1 Key Performance Indicators

**FIGURE 7:**
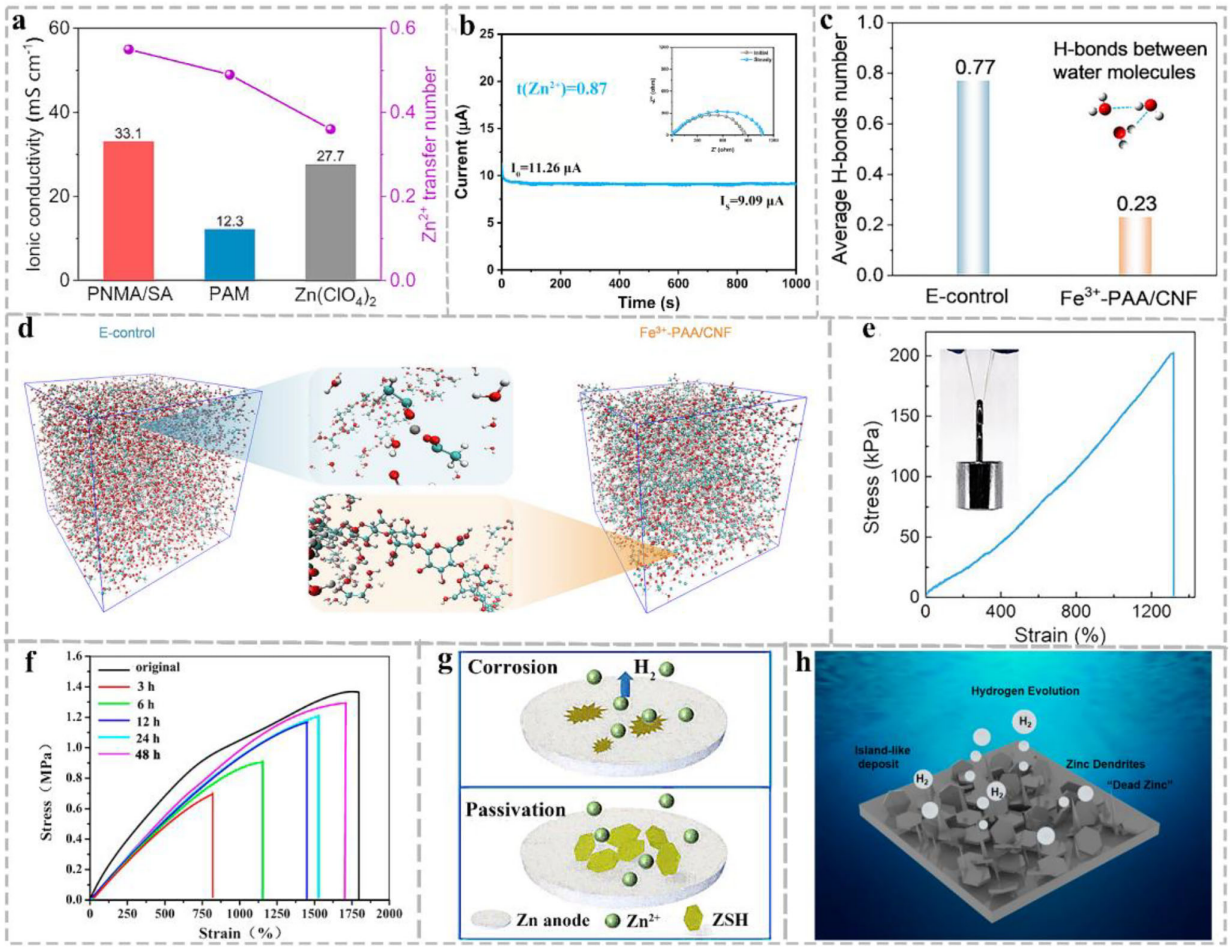
(a) Ionic conductivity of PNMA/SA, PAM, and Zn(ClO_4_)_2_ electrolytes. Reproduced with permission [119]. Copyright 2024, American Chemical Society. (b) Chronoamperometry profiles and Nyquist plots of Zn||Zn symmetric cells with DCH‐S electrolyte. Reproduced with permission [144]. Copyright 2025, Wiley‐VCH GmbH. (c) Average numbers of H‐bonds formed between water molecules obtained by MD simulation. (d) MD simulation snapshots of E‐control electrolyte and Fe^3+^‐PAA/CNF hydrogel electrolyte. Reproduced with permission [65]. Copyright 2025, Wiley‐VCH GmbH. (e) Stress–strain curves of the PAM‐PEGMA‐IL ionogel. Inset: Photograph of the PAM‐PEGMA‐IL ionogel lifting a 100 g weight. Reproduced under the terms of the Creative Commons CC BY license [66]. Copyright 2024, Advanced Science published by Wiley‐VCH GmbH. (f) Stress–strain curves of the original and self‐healed gels at various healing times. Reproduced with permission [85]. Copyright 2017, American Chemical Society. (g) Diagram of zinc anode interface corrosion reaction (up) and passivation reaction (down). Reproduced under the terms of a Creative Commons Attribution CC BY 4.0 license [149]. Copyright 2025, Di Zhang et al. (h) Schematic diagram of side reactions at the zinc anode interface. Reproduced under the terms of the Creative Commons Attribution (CC BY) license [150]. Copyright 2025. Licensee MDPI, Basel, Switzerland.

Ionic conductivity (σ) is a key performance metric for gel electrolytes, directly determining the power output characteristics of the battery. Its value can be quantitatively determined using the formula σ=h/(R·A), where h is the electrolyte thickness, R is the measured bulk resistance, and A is the effective contact area between the electrode and the electrolyte [30, 90]. Current research indicates that gel electrolytes with double‐network (DN) structures, such as a polyacrylamide/sodium alginate composite system, exhibit excellent ionic conduction [20, 27]. Their ionic conductivity can reach 33.1 mS·cm^−1^, significantly higher than the 27.7 mS cm^−1^ of conventional liquid electrolytes (Figure 7a). This enhancement is primarily attributed to the coordinated structures formed between the carboxylate groups on the sodium alginate chains and Zn^2+^ ions, which effectively promote directional zinc ion migration by constructing dynamic ion transport channels [119]. However, the ionic conductivity of most current gel electrolyte systems (ranging from 10^−9^ to 10^−3^ S·cm^−1^) still lags behind that of liquid electrolytes [39, 55]. This is particularly evident under low‐temperature conditions (e.g., −30°C), where the mobility of polymer chains is drastically reduced, blocking ion transport pathways and causing orders‐of‐magnitude decay in conductivity [72, 90]. Regarding ion transport mechanisms, t(Zn^2+^) is a crucial parameter measuring the contribution of Zn^2+^ to the total ionic current. Its value directly affects the charge/discharge efficiency and cycling stability of the battery. For instance, the DCH‐S gel system achieved a high t(Zn^2+^) of 0.87 (Figure 7b) by optimizing the polymer network structure. This effectively suppressed concentration polarization during cycling and blocked the pathways for zinc dendrite nucleation and growth [144]. In contrast, liquid electrolytes typically exhibit a lower t(Zn^2+^) (< 0.5) due to the lower migration resistance of anions. Gel electrolytes, however, can selectively impede anion migration through the electrostatic shielding effect of polymer chains, thereby establishing an ion transport mechanism dominated by Zn^2+^ conduction [30, 54, 145].

Optimizing the electrochemical stability window (ESW) of gel electrolytes requires the synergistic suppression of HER and electrode material corrosion [57, 146, 147]. Taking an antifreeze hydrogel system containing Fe^3+^‐carboxylate coordination structures as an example, it exhibits a wide ESW of 2.5 V at a low temperature of −20°C, significantly surpassing the 1.23 V typical of conventional aqueous electrolytes. This performance breakthrough primarily stems from the competing coordination of Fe^3+^ and Zn^2+^ for water molecules, which effectively reduces the activity of free water in the system, thereby inhibiting water‐splitting side reactions. MD simulations (Figure 7c,d) further corroborate this mechanism on a structural‐dynamics level. They reveal a significant reduction in the number of hydrogen bonds among water molecules and a decrease in free water activity within the Fe^3+^‐PAA/CNF system [65]. In the field of ionic liquid‐modified gels, incorporating functional gel electrolytes can effectively regulate zinc deposition behavior and suppress zinc dendrite growth. Their stable interface and ultrathin design help reduce internal battery resistance and minimize inactive volume, thereby significantly enhancing the energy density of zinc‐air batteries [30, 69, 71]. In contrast, solvent molecules in traditional liquid electrolyte systems are prone to decomposition reactions under high voltage or extreme potential conditions. For example, in a ZnSO_4_ aqueous solution, the hydrated [Zn(H_2_O)_6_]^2+^ ions undergo HER in the low‐potential region. This not only causes drastic fluctuations in electrolyte pH but also leads to the formation of a passivation layer on the electrode surface, ultimately resulting in irreversible degradation of battery performance [80, 148].

The mechanical properties of gel electrolytes critically determine the long‐term operational stability of flexible energy storage devices. The elastic modulus of the electrolyte must be compatible with the stress generated by the volume expansion of electrode materials during cycling to prevent mechanical debonding at the interface [50, 90, 151]. Regarding fracture resistance, the introduction of dynamic and reversible cross‐linking networks—including hydrogen bonds and metal‐coordination bonds—can significantly enhance material toughness. For instance, a PAM‐PEGMA‐IL ionogel system exhibits a tensile strength of 202 kPa and a fracture strain of 1318% (Figure 7e). Its energy dissipation mechanism, via localized stress dispersion, effectively inhibits crack initiation and propagation [66]. Self‐healing capability serves as another vital evaluation metric. Taking the PAA‐CNF‐Fe^3+^ gel as an example, this material can achieve a mechanical property recovery rate of 85% after 48 h of autonomous healing at 25°C (Figure 7f). This excellent self‐healing characteristic originates from the reversible coordination bonds formed between Fe^3+^ ions and carboxylate groups [85]. In contrast to liquid electrolyte systems that rely on separators for structural support, gel electrolytes synergistically integrate mechanical reinforcement and ionic conduction functions by constructing three‐dimensional polymer networks, such as cellulose nanofiber‐reinforced structures. This provides a novel material solution for the reliable operation of flexible electronic devices.

A fundamental performance trade‐off between ionic transport efficiency and mechanical stability is commonly observed in current gel electrolyte systems [30, 63]. Specifically, while increasing the polymer cross‐linking density can significantly enhance the material modulus—for instance, achieving a tensile storage modulus of 1000 kg·cm^−2^ for PVDF‐HFP‐based gels—an excessively dense network formed by over‐crosslinking severely hinders ion migration pathways, leading to a decline in ionic conductivity [90]. Regarding electrode‐electrolyte interfacial stability, existing systems generally suffer from compatibility defects, which can induce non‐uniform zinc ion deposition. As illustrated in the schematic diagrams of the zinc anode interface reactions in Figure 7g,h, dendritic deposits and electrochemically inactive “dead zinc” regions appear on the zinc anode surface [149, 150]. Conversely, gel electrolytes derived from metal‐organic frameworks (MOFs) exemplify a promising approach. Their unique hollow tubular structures allow for precise regulation of ion transport flux [116]. For example, a UiO‐66@PDA modified gel electrolyte (or ZIF‐90@PDA GPE) combines the porous MOF with the adhesive properties of polydopamine, forming a biomimetic mesh structure. This design not only improves mechanical strength but also achieves a high ion transference number by anchoring anions, demonstrating the significant advantage of structural engineering in enhancing overall battery performance [90]. Through ingenious porous structure design and interface modification, effective pathways are provided for the synergistic optimization of ion transport kinetics and mechanical/interfacial stability. This underscores the critical role of microstructure engineering in overcoming traditional performance bottlenecks.

### 4.2 Battery‐Level Performance

**FIGURE 8:**
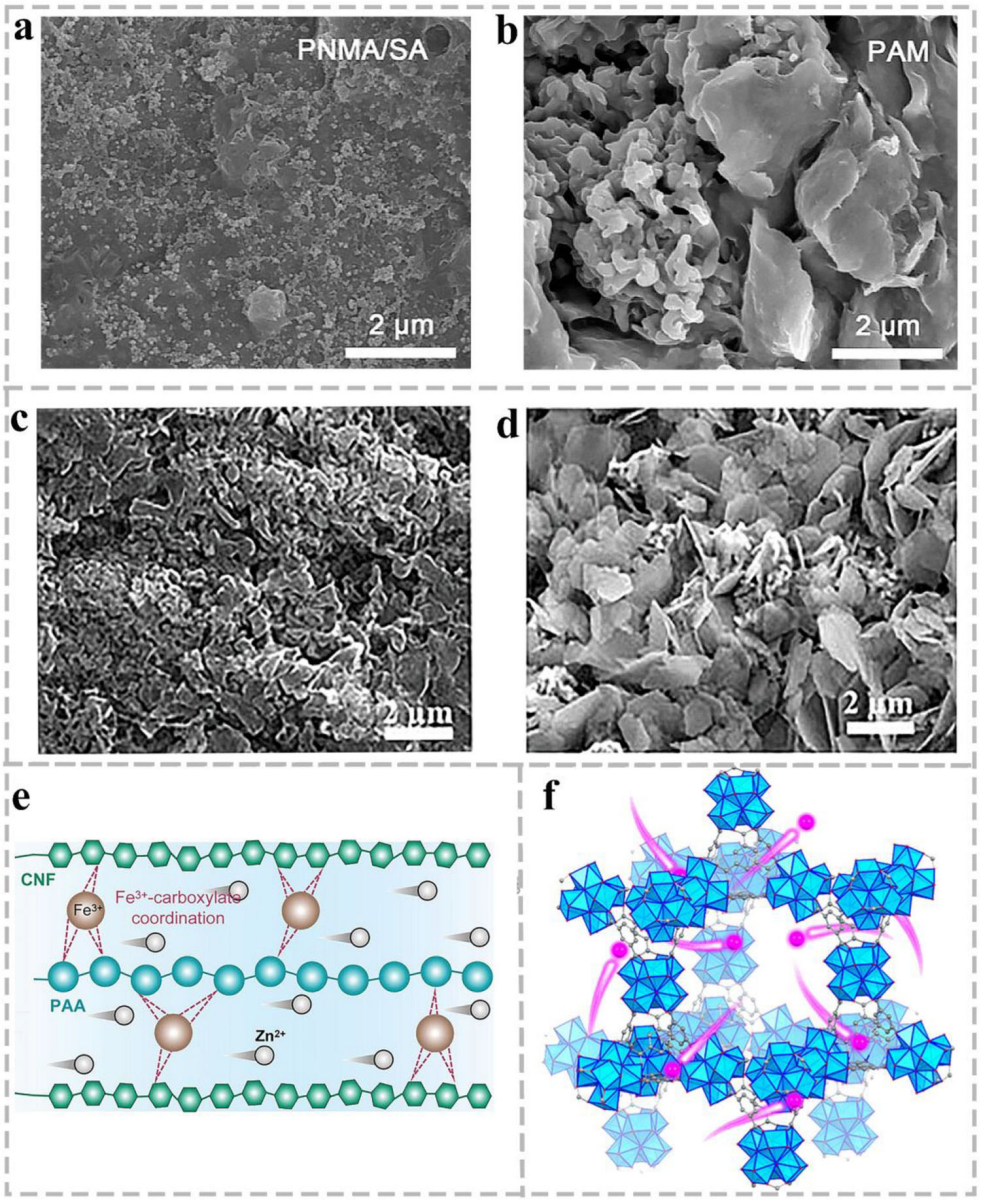
(a) SEM images of Zn anodes after cycling in symmetric Zn batteries with PNMA electrolyte. (b) SEM images of Zn anodes after cycling in PAM electrolyte symmetric Zn batteries. Reproduced with permission [119]. Copyright 2024, American Chemical Society. (c, d) SEM images for Zn anodes after plate/strip cycles for Zn‐MFZS (c) and 2 M ZnSO_4_ (d). Reproduced under the terms of the Creative Commons Attribution‐NonCommerical license 4.0 (CC‐BY‐NC) [153]. Copyright 2023, exclusive licensee American Association for the Advancement of Science. (e) Schematic diagram of the micro three‐dimensional network of PAA‐CNF‐Fe^3+^ composite hydrogel. Reproduced with permission [65]. Copyright 2025, Wiley‐VCH GmbH. (f) Crystal structure of ZnMOF‐808. Blue polyhedrons represent Zr─O clusters and Zn^2+^ ions are highlighted by pink balls. Reproduced with permission [154]. Copyright 2018, Elsevier Ltd. All rights reserved.

The cycling durability of gel electrolytes serves as a critical parameter for assessing their potential for engineering applications [118, 146, 152]. Current research demonstrates that ultrathin gel electrolytes, such as a TA‐SA composite system with a thickness of 24 µm, exhibit stable charge/discharge capability for over 3000 h in Zn||Zn symmetric cell tests. The charge/discharge voltage polarization is consistently maintained within 70 mV, significantly outperforming the cycle life of conventional liquid electrolytes (typically below 500 h). This superior cycling stability primarily originates from the precise regulation of zinc deposition kinetics by the gel matrix. For example, a PNMA/SA double‐network gel achieves uniform distribution of Zn^2+^ flux by constructing three‐dimensional ion transport channels. This enables a Zn||Zn symmetric cell to cycle for 2000 h at 1 mA·cm^−2^ without dendrite formation, as shown in the comparative images in Figure 8a,b [119]. In terms of structural innovation, a cellulose‐based gradient hydrogel elevates the Zn^2+^ transference number to 0.6 through a selective ion permeation mechanism. This system maintains a 98% capacity retention rate after 1900 h of cycling [30]. In contrast, liquid electrolyte systems typically struggle to exceed 200 h of cycle life due to persistent side reactions like HER and ZnO passivation layer formation, accompanied by significant capacity fade. SEM imaging further reveals (Figure 8c,d) that in aqueous electrolytes, the zinc anode surface suffers from severe formation of ZnO, Zn(HSO_4_)_2_, and zinc dendrites. Conversely, the surface of anodes in Zn‐MFZS SIEs remains dense even after 1000 h, with only minor amounts of ZnO and dendrites observed [30, 79, 153].

The thickness of the gel electrolyte significantly impacts the energy density metrics of the full cell. According to existing studies, reducing the electrolyte layer thickness from 200 to 30 µm can increase the gravimetric energy density by 40% to 523 Wh·kg^−1^, while the volumetric energy density reaches 1609 Wh·L^−1^. This performance enhancement is primarily attributed to the reduced proportion of inactive components. For instance, in a PVDF‐HFP‐based gel electrolyte, reducing the thickness to 50 µm results in a 35% increase in cell‐level energy density compared to a traditional 200 µm‐thick gel system. This improvement is due to the more effective utilization of active materials [90]. Regarding power characteristics, a PAM‐SC polarized gel electrolyte demonstrates excellent high‐rate performance. It maintains a discharge specific capacity of 136.2 mAh·g^−1^ even at a high current density of 8 mA·cm^−2^. This performance is mainly attributed to its high ionic conductivity of 324.68 mS·cm^−1^ and low interfacial resistance (<10 Ω·cm^2^). These properties work synergistically to reduce internal cell resistance and enhance charge transfer efficiency [60, 146].

Flexible energy storage devices must maintain stable electrochemical performance under complex mechanical deformation. For instance, a PVA‐GO‐based fibrous battery retains over 95% of its discharge capacity after 1000 bending cycles at 180°, and maintains a 92% energy conversion efficiency even under 100% tensile strain [155]. This excellent deformability primarily stems from the three‐dimensional elastic network structure of the gel electrolyte (Figure 8e). An example is the PAA‐CNF‐Fe^3+^ composite hydrogel, which exhibits an ultimate fracture strain of 1318% and a tensile modulus of 202 kPa, enabling effective adaptation to various complex deformation scenarios [65]. Regarding textile‐integrated applications, a Zn‐air sleeve‐type battery demonstrates a capacity retention of 93.62% after 5000 charge/discharge cycles under 50% tensile strain. Furthermore, the power density in a bent state only decreased by 7% (from 57.5 to 53.4 W·kg^−1^), fully demonstrating the suitability of gel electrolytes for wearable devices [30, 137, 156, 157].

Current flexible battery technologies face a persistent dilemma between enhancing energy density and optimizing mechanical reliability. Specifically, while ultrathin gel electrolyte designs (thickness <30 µm) can effectively increase the system's energy density, their resistance to dendrite penetration is significantly compromised. For example, a PAM‐based gel electrolyte suffered short‐circuit failure due to zinc dendrite puncture after only 1330 h of cycling [22, 30, 72]. In contrast, MOF‐functionalized modified gels exemplify a promising structural engineering approach. Their unique hollow tubular structures enable precise regulation of Zn^2+^ transport flux (Figure 8f) [154]. This design allows a Zn/MnO_2_ full cell to maintain a discharge specific capacity of 150 mAh·g^−1^ after 1000 cycles at a 5 C rate, demonstrating the effectiveness of structural engineering in overcoming the performance bottlenecks of flexible batteries [50]. For FZABs, gel electrolyte design must simultaneously address multiple requirements: ionic conduction, dendrite suppression, mechanical flexibility, and air cathode interface stability. By drawing inspiration from the structural modulation strategies of functional materials like MOFs, and by focusing on constructing adaptive interfaces and three‐dimensional ion‐conducting networks, it holds promise to unify high energy density with long‐term cycling stability. This integration offers a potential pathway to advance the practical application of flexible energy storage devices.

### 4.3 Existing Challenges and Trade‐Offs

Although ultra‐thin gel electrolytes (thickness <30 µm) can effectively increase battery energy density, the consequent sharp rise in interfacial contact resistance has become a core bottleneck limiting further performance breakthroughs. Existing data indicate that when the electrolyte thickness is reduced from 200 to 24 µm, the interfacial impedance increases by over 50%, primarily due to the reduction in the effective electrode‐electrolyte contact area [22, 90, 130]. For example, in a zinc symmetric cell using a PVDF‐HFP‐based ultrathin gel electrolyte, the initial polarization voltage is significantly higher (120 mV) compared to a liquid electrolyte system (50 mV), which can be attributed to poor interfacial contact [50, 72]. This phenomenon is mainly due to two mechanisms: first, insufficient physical adsorption between the polymer chains and the electrode surface; and second, the significantly weakened mechanical compliance of the ultra‐thin structure in accommodating electrode volume changes [158, 159, 160]. The trade‐off between decreasing gel polymer electrolyte thickness and maintaining mechanical strength presents a significant challenge: a 1 µm‐thick robust gel polymer electrolyte supported by an ultrathin polyethylene separator exhibits a tensile strength exceeding 400 MPa, whereas conventional gel polymer electrolytes without substrate reinforcement typically display large thicknesses and relatively high polymer matrix content to maintain mechanical integrity [161]. To address this issue, strategies such as employing in situ polymerization processes or introducing flexible functional layers (e.g., cellulose nanofiber composite structures) can optimize the interfacial impedance to below 10 Ω·cm^2^ [130, 151, 162]. Specifically, in situ photopolymerization of a PVDF‐HFP‐based gel polymer electrolyte onto an air cathode reduced the charge transfer resistance from 255.9 to 89.66 Ω and extended the cycle life from 110 h to 940 h, while in situ polymerization of a free‐standing gel polymer electrolyte with carbonyl–Li^+^ coordination achieved a tensile strength of ∼1.0 MPa and an elongation at break of ∼920% [163, 164]. However, these modification strategies often come with a substantial increase in fabrication complexity, necessitating a balance between performance enhancement and process cost.

The design of gel electrolyte systems inherently involves significant trade‐offs among ultra‐thinning, ionic transport efficiency, and structural mechanical performance [36, 63]. For instance, a PAM‐based gel electrolyte compressed to a thickness of 20 µm can achieve a high ionic conductivity of 41.5 mS·cm^−1^. However, this comes at the cost of its fracture strain significantly decreasing from 1318% to 600% and its elastic modulus dropping from 202 to 80 kPa [50, 66]. This inverse relationship between thickness reduction and mechanical deterioration is further corroborated by systematic studies on PAM hydrogels, where increasing electrolyte content to boost ionic conductivity invariably leads to a marked decline in mechanical properties; for example, pure PAM hydrogels exhibit intrinsic mechanical strength typically below 200 kPa, rendering them prone to structural collapse under compressive stress despite their high water absorption capacity [54]. This performance trade‐off phenomenon mainly originates from the dual regulation dilemma of the polymer network structure: increasing the crosslinking density can enhance mechanical stability, for example, the modulus of the PAA‐CNF‐Fe^3+^ composite gel reaches 500 kPa, but it will form a dense network that obstructs ion migration channels, causing the ionic conductivity to sharply drop to the magnitude of 10^−5^ S·cm^−1^ [85]. To address this conflict, multi‐scale structural optimization strategies have shown considerable promise. A notable example is a double‐network gel system with a gradient pore structure, such as one composed of tannic acid and sodium alginate (TA‐SA). Through the synergistic effect of dynamic hydrogen bonds and metal‐coordination bonds, this system maintains an ionic conductivity of 33.1 mS·cm^−1^ while simultaneously achieving a fracture strain of 150%. This demonstrates the successful co‐optimization of ion transport and mechanical properties [119, 124]. Similarly, a PVA‐PBDA@PAM double‐network hydrogel fabricated via a one‐pot blending method achieves a tensile strength of 0.6 MPa, exceptional stretchability of 1689%, and a high ionic conductivity of 9.84 mS·cm^−1^, surpassing previously reported borate‐based hydrogels and validating the efficacy of dual‐network architectures in resolving the ion transport‐mechanical robustness trade‐off [165]. In addition, Table 3 also summarizes the performance trade‑offs associated with representative gel electrolyte design strategies.

**TABLE 3:** Performance trade‐offs of representative gel electrolyte design strategies.

Design strategy	Representative system	Ionic conductivity (mS·cm^−1^)	Mechanical strength	Cycle life	Key trade‐off	Ref.
High crosslinking density	DCZ‐gel	38.6	Tensile stress: 2.08 MPa, strain: 145%; Compressive stress: 4.42 MPa, strain: 76%; Toughness: 1.20 MJ·m^−3^; Elastic modulus: 1.93 MPa	Zn||Zn: >2000 h (0.5 mA·cm^−2^/0.5 mAh·cm^−2^); >400 h (10 mA·cm^−2^/10 mAh·cm^−2^); Zn||PANI full cell: >2000 cycles at 2000 mA·g^−1^	Through optimization of the epichlorohydrin (ECH) dosage, a densely crosslinked network was established to inhibit dendrite puncture, while the hierarchical porous architecture was preserved to function as ionic pathways, avoiding the ion‐transport blockage often associated with high crosslink density. The elastic contribution from physical crosslinking (hydrogen bonds and crystalline regions), combined with the strengthening effect of chemical crosslinks, enabled a synergistic trade‑off between stiffness and ductility.	[147]
Nanofiller reinforcement	PAM‐SiO_2_ hydrogel electrolyte	350.2	Although the tensile strength is lower than that of pure PAM‐attributable to the disruption of uniformity by the porous structure‐it remains adequate to satisfy the flexing, tensile, and torsional demands of flexible batteries.	FZAB charge‐discharge cycling: 62.6 h (PAM‐0.1 g SiO_2_) vs. 43.7 h (pure PAM) at 2 mA·cm^−2^; 90° bending for 100 cycles‐maintained functionalities.	Although SiO_2_ nanofillers act as sacrificial pore‑formers to improve electrolyte absorption and ionic conduction, they inevitably undermine mechanical integrity due to pore‑induced structural imperfections and diminished crosslink density in the pore walls. A loading of 0.1 g was found to be optimal, whereas a higher amount (0.15 g), though slightly enhancing conductivity, resulted in inferior water retention and reduced cycle durability.	[64]
Ultrathin design	PPQAC membrane electrolyte	30.2	Tensile strength 4.6 MPa (dehydrated), Young's modulus 24.1 MPa; electrolyte‐saturated: reduced but flexible, sustaining 0°–180° bending.	ZAB cycling stability >100 h at 5 mA·cm^−2^; symmetric Zn||Zn cell ∼800 h at 10 mA·cm^−2^; round‐trip energy efficiency 59.8%	An ultrathin fiber membrane design reduces battery thickness and internal resistance, yet it necessitates simultaneous solutions to i) mechanical strength‐via reinforcement with a PL polysaccharide cellulose scaffold and GA crosslinking; ii) ionic conductivity‐via QA quaternary ammonium groups as OH^−^ carriers; and iii) electrolyte retention‐via the porous fibrous network with high uptake (360%) and retention (78%/40 h). This fibrous interwoven architecture, in contrast to traditional bulk gels, achieves a synergy among thinness, flexibility, and conductivity.	[68]
Double‐network structure	PVA/N‐PAM/KOH alkaline gel polymer electrolyte (AGPE)	309.9	Tensile strength 498.4 kPa (8 mol·L^−1^ KOH), Young's modulus 284.7 kPa, toughness 0.69 MJ·m^−3^; breaking elongation 237.85%	Zn‐air battery: 113 cycles (2.0 mA·cm^−2^); energy efficiency 70.2%; stable discharge 675 min at 2.0 mA·cm^−2^; functional under twisting, bending (90°/150°), and fracture conditions.	The hybrid dual‑network, comprising a PAM covalent network and a PVA microcrystalline physical network, was constructed. The former ensures excellent electrolyte absorption, water retention, and ionic conduction pathways, whereas the latter contributes to mechanical robustness and energy dissipation. In addition, the hydrogen‑bond network promotes OH^−^ migration through the Grotthuss mechanism in a synergistic manner. The optimum KOH concentration was found to be 8 mol·L^−1^; beyond this value (e.g., 10 mol·L^−1^), hydrogen bonding is disrupted, leading to a deterioration in mechanical strength.	[169]
Water‐in‐salt/gel	WIS‐PAA/CNF nonalkaline gel polymer electrolyte	1.63	Breaking strength 22.3 kPa, elongation 770% (WIS‐PAA/CNF); 18.2 kPa / 618% (WIS‐PAA); CNF reinforcement via physical entanglement and hydrogen bonding.	ZAB ultra‐long cycling 1300 h (3545 cycles, 0.1 mA·cm^−2^, 22 min/cycle in ambient air); 700 h (344 cycles, 122 min/cycle); energy efficiency 59.5%; SIW‐PAA failed at 400 h, PAA/KOH at 25 h.	Although the water‑in‑salt (WIS) approach effectively lowers water activity to inhibit hydrogen evolution, zinc corrosion, and carbon dioxide absorption, it significantly compromises ionic conductivity (dropping from 98.5 to 1.63 mS·cm^−1^). To mitigate this trade‑off, a cellulose nanofiber (CNF) dual‑network is introduced to compensate for the mechanical deterioration, and dynamic water adsorption–desorption with the ambient environment serves to replenish water loss. In addition, the non‑alkaline nature of the system prevents the generation of Zn(OH)_4_^2−^ and carbonate species, and the reversible O_2_/ZnO_2_ redox chemistry underpins an exceptionally prolonged cycling stability.	[35]
Janus/gradient structure	LDH/CMC/PAM dual‐network hydrogel	145.93	0.19 MPa (stress); 530% strain.	160 h (@1 mA·cm^−2^); 90 h (@5 mA·cm^−2^, bent)	The Janus strategy, through the synergy between the Grotthuss mechanism in the LDH interlayers and the Vehicle mechanism of the CMC/PAM dual network, simultaneously enhances ionic conductivity, mechanical strength, and cycling life. This approach effectively overcomes the conventional trade‑off dilemma of gel electrolytes, which typically suffer from either high conductivity with short lifespan or high strength with low conductivity.	[138]

The industrialization of current precision thin‐film fabrication techniques, such as electrospinning and spin‐coating, is constrained by dual limitations of low yield and insufficient cost‐effectiveness [130]. For example, producing electrospun PVA/PAA nanofiber membranes requires stringent control of environmental humidity (±5% RH) and temperature (25 ± 1°C). This results in a single‐batch product qualification rate of only 60%‐70%, accompanied by high equipment energy consumption of up to 50 kWh·m^−2^ [166]. Notably, ultra‐thin gel systems exhibit high sensitivity to compositional purity. Studies indicate that a fluctuation of more than 5% in Zn^2+^ concentration can lead to about a 30% decrease in ionic conductivity [30, 144, 167]. In the context of scaled‐up manufacturing, solvent casting demonstrates the lowest cost advantage, but its thickness control precision is limited to ±15 µm. In contrast, atomic layer deposition (ALD) technology can achieve thickness precision at the ±1 nm level, albeit with a significant increase in cost per unit area [29, 158, 168]. Future technological breakthroughs should focus on developing low‐cost dry processes (e.g., hot‐pressing) or innovative hybrid processes (such as coupling electrospinning with spray coating). The goal is to achieve a balance between performance metrics and manufacturing costs through the synergistic optimization of multiple techniques.

## 5 Design Strategies for Gel Electrolytes Targeting Dendrite Suppression and Ultrathin Design

To overcome the challenges of excessive dendrite growth and interfacial degradation at the zinc anode while meeting the urgent demand for ultrathin, high‐energy‐density power sources in flexible electronics, researchers have proposed a series of innovative gel electrolytes designs. These designs are based on multi‐scale structural engineering, aiming for the systematic enhancement of overall electrolyte performance through synergistic approaches. These include optimizing ion transport pathways, reinforcing mechanical stability and interfacial compatibility, suppressing parasitic side reactions, and constructing novel architectures.

### 5.1 Regulate Ion Transport to Achieve Uniform Deposition

**FIGURE 9:**
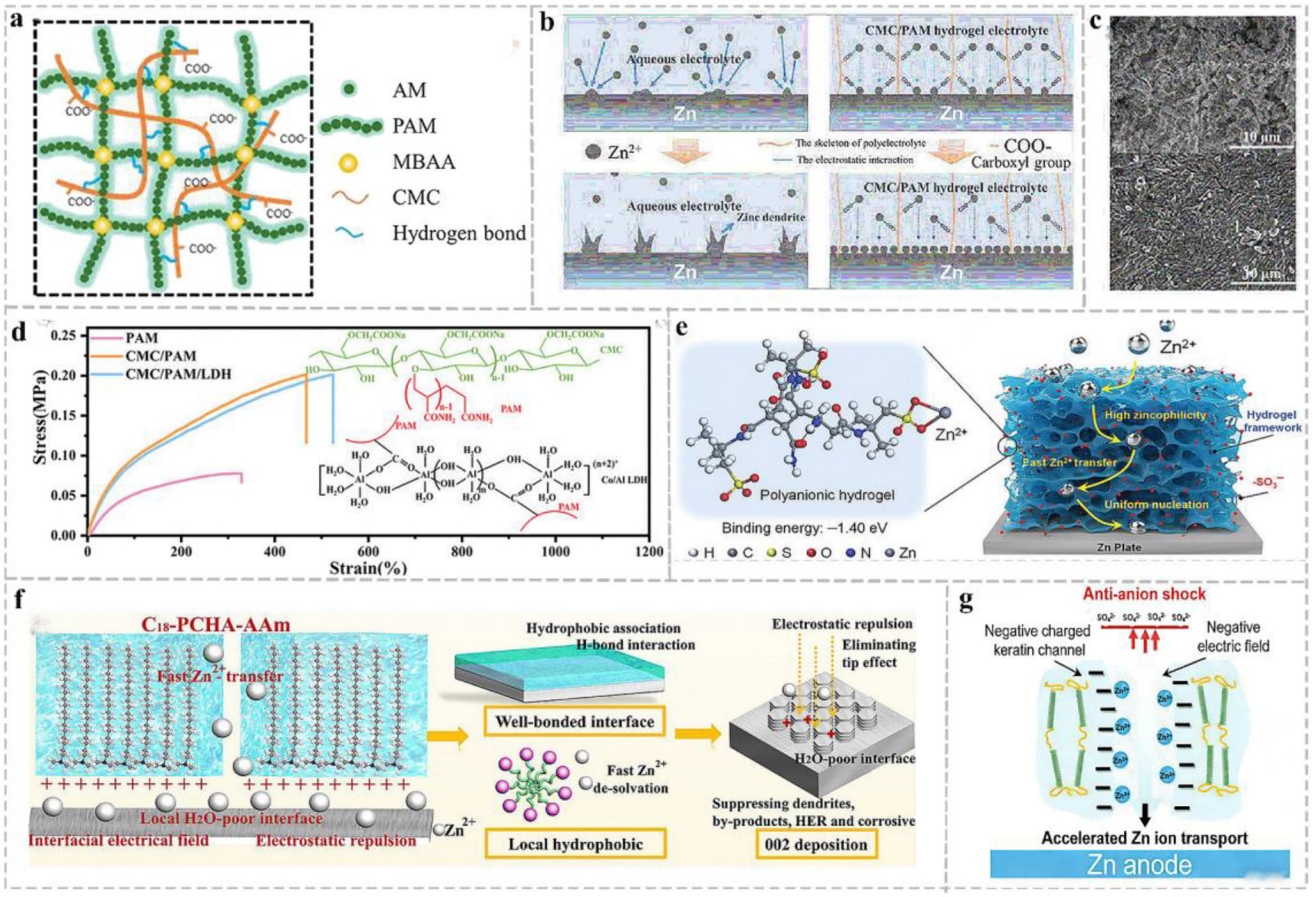
(a) Illustration of the double‐network of CMC/PAM hydrogel. (b) mechanism of inhibition of dendritic growth by CMC/PAM HE, and (c) SEM images of zinc anodes with PAM HE (up) and CMC/PAM HE (down). Reproduced with permission [29]. Copyright 2024, Wiley‐VCH GmbH. (d) Stress–strain curves at a 10 mm·min^−1^ stretching rate for PAM, CMC/PAM, and LDH/CMC/PAM hydrogels. Reproduced with permission [138]. Copyright 2024, Wiley‐VCH GmbH. (e) Optimized Zn^2+^ adsorption configuration on the ‐SO_3_^−^ group. Reproduced with permission [175]. Copyright 2022, Wiley‐VCH GmbH. (f) Schematic illustrations of the polycationic hydrogel electrolyte and its regulation of Zn deposition behavior. Reproduced with permission [176]. Copyright 2024, Royal Society of Chemistry. (g) Schematic of the anti‐anion shock effect on Zn‐ion transport triggered by negatively charged keratin.Reproduced with permission [177]. Copyright 2022, Wiley‐VCH GmbH.

As illustrated in the upper‐left quadrant of Figure 2, regulating ion transport to achieve uniform zinc deposition represents one of core design strategies for gel electrolytes in FZABs. Uniform zinc deposition is a prerequisite for achieving a long‐cycle‐life zinc anode, and the ion transport behavior within the gel electrolyte is key to determining the deposition morphology. By optimizing the topological structure of the polymer network and its functional modification, the transport pathways and migration kinetics of Zn^2+^ can be precisely regulated, thereby effectively suppressing abnormal dendrite growth [30, 43, 144]. A core strategy involves constructing a three‐dimensional polymer framework with combined physical and chemical cross‐linking, which can form homogeneous ion transport channels [7]. For instance, polymer systems such as PAM, PVA, and CMC can be employed to build gel electrolytes with diverse network architectures [100, 170]. In particular, DN hydrogels, formed via interpenetrating networks of chemically cross‐linked PAM and physically entangled PVA/CMC, demonstrate significant performance advantages [72]. As shown in Figure 9a, during the synthesis of a PAM/CMC DN hydrogel, the chemically cross‐linked PAM skeleton and the physically entangled CMC fibers form an interpenetrating structure, providing directional migration pathways for Zn^2+^ [29]. In such composite systems, networks with different cross‐linking mechanisms act synergistically: the rigid network provides mechanical strength, while the flexible network forms well‐defined nanochannels. These channels not only restrict the lateral expansion of dendrites via a steric hindrance effect but, more importantly, construct directional pathways for Zn^2+^ migration. This ensures a uniform distribution of ion flux across the electrode surface, thereby eliminating the localized current overload caused by the “tip effect” [171, 172]. Taking a CMC/PAM composite hydrogel electrolyte as an example, it can achieve a stretchability of up to 2700% while maintaining a high ionic conductivity of 6.37×10^−2^ S·cm^−1^. This validates that an optimized polymer network can homogenize ion flux without compromising ionic conduction efficiency [173]. Furthermore, characteristic functional groups on the polymer chains (e.g., the carboxylate ─COO^−^ groups in CMC and the hydroxyl ‐OH groups in PVA) can dynamically interact with Zn^2+^ via weak coordination [4, 31]. This interaction mechanism can lower the activation energy for ion migration and induce the formation of two‐dimensional layered zinc deposits rather than three‐dimensional dendritic structures [146, 174]. As illustrated in Figure 9b,c, the ─COO^−^ groups in CMC can effectively adsorb Zn^2+^ through electrostatic interactions, guiding uniform zinc ion deposition and inhibiting dendrite formation. SEM observations further corroborate the effectiveness of this mechanism: the zinc anode surface using the DN hydrogel electrolyte appears smooth and compact without obvious dendrites, whereas numerous 3D dendritic structures are observed in traditional liquid electrolytes [29]. Additionally, this DN hydrogel electrolyte itself exhibits excellent mechanical properties. As shown in Figure 9d, the stress‐strain curve of the DN hydrogel demonstrates significantly higher toughness compared to single‐network systems. This enhanced toughness helps accommodate the volume changes of the electrode during cycling, maintaining the integrity and stability of the interface structure [138].

Another pivotal strategy focuses on increasing the zinc ion transference number (t(Zn^2+^)), which represents the fraction of current carried by Zn^2+^ within the electrolyte [4, 26]. It is quantitatively defined by the following formula:

(6) $$t\left(Zn^{2+}\right)=\frac{\sigma_{Zn^{2+}}}{\sigma_{total}}=\frac{D_{Zn^{2+}}\cdot c_{Zn^{2+}}+z_{Zn^{2+}}^{2}}{\sum_{i}D_{i}\cdot c_{i}\cdot z_{i}^{2}}$$

where *σ*, *D*, *c*, and *z* denote partial conductivity, diffusion coefficient, concentration, and charge number, respectively; experimentally, it is determined by the Bruce‐Vincent method using

(7) $$t\left(Zn^{2+}\right)=\frac{I_{ss}\left(\Delta V-I_{0}R_{0}\right)}{I_{0}\left(\Delta V-I_{ss}R_{ss}\right)}$$

in symmetric Zn||Zn cells. Where *I_0_* and *I_ss_* are the initial and steady‐state currents, respectively; *R_0_* and *Rss* are the initial and steady‐state interfacial resistances, respectively; and *ΔV* is the applied DC polarization voltage. In conventional liquid electrolytes, t(Zn^2+^) typically ranges from 0.2 to 0.5 due to the lower migration resistance of anions compared to heavily solvated Zn^2+^ cations, which leads to anion‐dominated charge transport and severe concentration polarization at the electrode interface [107, 178, 179]. A t(Zn^2+^) value exceeding 0.5 is considered the critical threshold for effectively suppressing concentration polarization, as it ensures Zn^2+^ becomes the majority charge carrier; advanced gel electrolytes incorporating polyanionic frameworks or zwitterionic architectures have achieved t(Zn^2+^) values of 0.69‐0.91, fundamentally homogenizing ion flux and inhibiting dendrite nucleation [51, 180, 181, 182].

In conventional electrolyte systems, t(Zn^2+^) is typically low (e.g., <0.5), indicating that current is predominantly carried by the migration of anions such as SO_4_^2−^ or Cl^−^ [146, 183]. This charge transport mechanism presents a significant issue during zinc deposition: Zn^2+^ is continuously consumed at the anode interface, leading to a sharp concentration drop, while anions accumulate, establishing a concentration gradient. In turn, triggers concentration polarization [80, 84, 184]. As one of the primary thermodynamic driving forces for dendrite growth, concentration polarization exacerbates the inhomogeneous distribution of ions at the electrode surface. To address this challenge, researchers have developed polyanionic gel electrolyte systems that incorporate structural regulation via fixed charged groups on the polymer backbone. The sulfonate group (─SO_3_^−^) has emerged as a preferred functional group due to its strong zinc affinity and stable negative charge [30, 56, 185]. By grafting sulfonate groups onto polymer chains (e.g., sulfonated poly (ether ether ketone) SPEEK or modified cellulose), the immobilized ─SO_3_^−^ groups restrict the migration of free anions via electrostatic repulsion while simultaneously creating preferential transport pathways for Zn^2+^ [30, 186]. As shown in Figure 9e, the optimized DFT‐calculated adsorption configuration of Zn^2+^ on an ─SO_3_^−^ group (binding energy ‐1.40 eV) confirms their strong interaction. Figure 9f further illustrates how a polyanionic hydrogel electrolyte regulates zinc deposition behavior, wherein the immobilized ─SO_3_^−^ groups repel free anions and construct Zn^2+^‐selective channels to enhance the transference number. This design leads to a marked improvement in t(Zn^2+^). Experimental data indicate that sulfonate modification can elevate t(Zn^2+^) from around 0.4 to 0.69, with further optimization via gradient structures achieving values above 0.80 [30], some polyanionic hydrogel electrolytes have even realized an ultra‐high t(Zn^2+^) of 0.912 [187]. A high t(Zn^2+^) system effectively mitigates concentration polarization by maintaining ionic concentration equilibrium at the electrode interface, thereby thermodynamically suppressing mass‐transfer‐controlled dendrite growth. DFT calculations further reveal that the strong interaction between sulfonate groups and Zn^2+^ not only promotes uniform ion distribution but also, through a characteristic “paddle‐wheel mechanism” dynamically accelerates Zn^2+^ diffusion via group rotation. This significantly reduces interfacial charge transfer resistance, ultimately leading to dense and uniform zinc deposition [30, 188, 189]. As shown in Figure 9g, the “anti‐anion‐shock” effect triggered by negatively charged keratin illustrates how the directional migration of Zn^2+^ under an electric field reduces concentration polarization and inhibits dendrite growth [30]. For FZABs applications, developing gel electrolytes that concurrently possess a high zinc ion transference number and excellent mechanical robustness is crucial. Constructing efficient ion‐selective transport channels through strategies such as employing polyanionic frameworks not only fundamentally suppresses zinc anode dendrites but also provides a stable three‐phase reaction interface for the air cathode. This represents a key pathway toward realizing FZABs with high cycling stability and high energy density.

### 5.2 Enhance Mechanical and Interfacial Stability to Resist Dendrites

As depicted in the upper‐right quadrant of Figure 2, enhancing mechanical and interfacial stability to construct robust physical barriers constitutes another core design strategy. Based on the electrochemical homogenization of zinc deposition behavior (as detailed in Section 5.1), which focuses on optimizing ion transport kinetics, further enhancing the mechanical properties and interfacial compatibility of gel electrolytes emerges as a critical strategy for constructing a robust physical barrier system to fundamentally suppress dendrite penetration. An ideal gel electrolyte system needs to fulfill dual functionalities. First, by forming a three‐dimensional network structure with high mechanical strength, it must establish a physical barrier capable of directly inhibiting the vertical growth of zinc dendrites [92, 120]. Second, it should facilitate the formation of a stable, dynamically self‐healing interlayer at the electrode/electrolyte interface. This interlayer must adapt to the volume fluctuations of the zinc anode during cycling, mitigating interfacial stress to suppress the formation of dendrite nucleation sites and block the lateral propagation paths of any existing dendrites [144]. This synergistic mechanism, combining mechanical blocking and interfacial modulation, provides a theoretical foundation for achieving highly safe zinc‐metal batteries [30].

**FIGURE 10:**
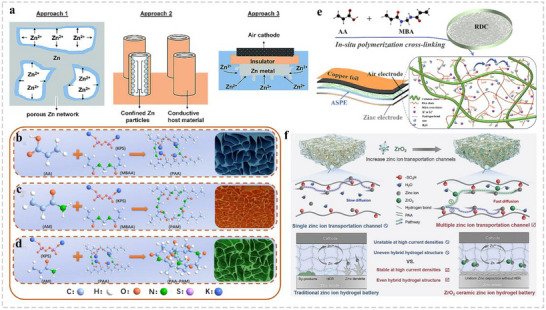
(a) Approaches to minimize zinc‐dendrite growth and undesired relocation: approach 1, employing a high surface area porous zinc structure; approach 2, confining zinc in 3D conductive host materials; approach 3, direct physical inhibition/ suppression. Reproduced with permission [3]. Copyright 2018, WILEY‐VCH Verlag GmbH & Co. KGaA, Weinheim. The crosslinking route of the structure of the polymer gel electrolytes and the corresponding chemical structure: (b) PAA polymer network; (c) PAM polymer network; (d) PAA‐PAM polymer dual‐network. Reproduced with permission [36]. Copyright 2025, Elsevier B.V. All rights are reserved. (e) Schematic depiction of the RDC/N‐PAA/KOH polyelectrolyte. Reproduced with permission [29]. Copyright 2024, Wiley‐VCH GmbH. (f) Schematic comparison of conventional hydrogel electrolytes and zirconia‐modified hydrogels: highlighting zinc‐ion transport pathways, associated challenges in traditional batteries, and the improved ion guidance and uniform deposition achieved with zirconia incorporation. Reproduced with permission [192]. Copyright 2025, Wiley‐VCH GmbH.

Enhancing the mechanical properties of gel electrolytes represents a direct and effective approach to constructing physical barrier layers and suppressing zinc dendrite growth [26, 55, 73]. According to electro‐chemo‐mechanical coupling theory, when the shear modulus of an electrolyte material significantly exceeds a critical value of the deposited metal, an effective mechanical suppression effect is established, thereby impeding the vertical propagation of dendrites. Although achieving the ideal solid‐state electrolyte property of a GPa‐level modulus remains a significant technical challenge in hydrogel systems, recent studies confirm that optimizing the elastic modulus to the MPa range (e.g., 1–10 MPa) can already significantly delay dendrite penetration through stress buffering mechanisms [74, 190]. As illustrated in Figure 10a, gel electrolytes can effectively inhibit the penetration and growth of zinc dendrites by constructing a mechanical barrier. The primary design strategies can be categorized into three types: (i) constructing high‐surface‐area porous zinc structures (Method 1) to reduce local current density and guide uniform deposition; (ii) introducing three‐dimensional conductive host materials (Method 2) to provide uniform nucleation sites and spatial support for zinc deposition [26, 55]; and (iii) employing direct physical suppression/blocking layers (Method 3) to mechanically constrain outward dendrite growth [3]. This schematic further visually contrasts dendrite growth behavior in conventional liquid electrolytes versus mechanically reinforced gel electrolytes, revealing how gel systems with MPa‐level moduli effectively suppress dendrite penetration through stress buffering and spatial confinement [110, 143]. To achieve this modulus target, DN hydrogel designs and nanocomposite reinforcement strategies have become mainstream technical pathways [43]. For constructing DN systems, taking the interpenetrating PAA‐PAM network as an example, the topological interpenetration of a rigid first network (highly chemically cross‐linked) and a flexible second network (physically entangled) achieves a synergistic enhancement of modulus and toughness [170]. As shown in Figure 10b–d, the structures of a PAA single network, a PAM single network, and their interpenetration forming the PAA‐PAM DN are presented. The construction of the DN embodies a “rigid skeleton and flexible entanglement” synergistic enhancement design philosophy. The corresponding microstructural images on the right clearly show the multi‐scale interpenetrating porous structure formed by this system, characterized by a combination of macropores and micropores, which significantly increases the overall material porosity [36]. Here, the first network serves as a structural skeleton providing foundational modulus support, while the second network effectively inhibits crack propagation through energy dissipation mechanisms such as chain slippage and reversible bond breaking, endowing the gel system with excellent puncture resistance [30]. The nanocomposite reinforcement strategy focuses on incorporating high‐modulus nanofillers, including carbon nanotubes (CNTs), zirconium oxide (ZrO_2_) nanoparticles, and cellulose nanocrystals. By forming strong interfacial interactions with the polymer matrix, these nanofillers construct a three‐dimensional stress transfer network, significantly enhancing the composite gel's elastic modulus and fracture energy [62, 85]. As shown in Figure 10e, the construction of a DN gel electrolyte first involves complete infiltration of acrylic acid (AA) monomers into a regenerated degreased cotton (RDC) cellulose network, followed by in‐situ polymerization in the presence of an initiator and cross‐linker, ultimately yielding an RDC/N‐PAA/KOH‐based composite polymer electrolyte. This design forms a DN structure with nanocomposite features, where nanofibers establish a three‐dimensional percolating network within the polymer matrix. Arrows in the figure clearly indicate the resulting stress transfer pathways [29, 191]. This structure significantly improves the overall modulus by enhancing interfacial interactions between nanofillers and the polymer matrix, thereby constructing an effective 3D stress transfer network. Experimental data indicate that adding a small amount of ZrO_2_ nanoparticles (mass fraction <5%) can increase the hydrogel modulus by an order of magnitude, forming a mechanical barrier capable of effectively resisting physical puncture by zinc dendrites, consequently leading to a significant improvement in battery cycle life [192]. As shown in Figure 10f, the zinc ion transport behavior and deposition morphology in a conventional hydrogel are compared with those in a ZrO_2_‐modified hydrogel. The left image reveals dendrite generation and penetration in the unmodified gel, while the right image shows that uniformly dispersed ZrO_2_ nanoparticles can guide ordered zinc deposition, visually validating the conclusion that “a small amount of inorganic filler can significantly enhance gel modulus and suppress dendrites” [30]. This modification mechanism can be further explained at the molecular level: due to the strong polarity of the ZrO_2_ surface, adsorbed water molecules readily dissociate, forming abundant ‐OH groups. These groups, on one hand, effectively suppress the hydrogen evolution side reaction during cycling through interactions with the hydrogel chains; on the other hand, the Zr‐OH bonds can act as Lewis acid sites, weakening the solvation interaction between Zn^2+^ and anions, thereby promoting the desolvation process of Zn^2+^ and ultimately enhancing the ionic conductivity of the system [30, 192, 193]. This strategy of optimizing mechanical properties through multi‐scale structural regulation provides an important material design paradigm for developing high‐safety zinc‐metal batteries.

**FIGURE 11:**
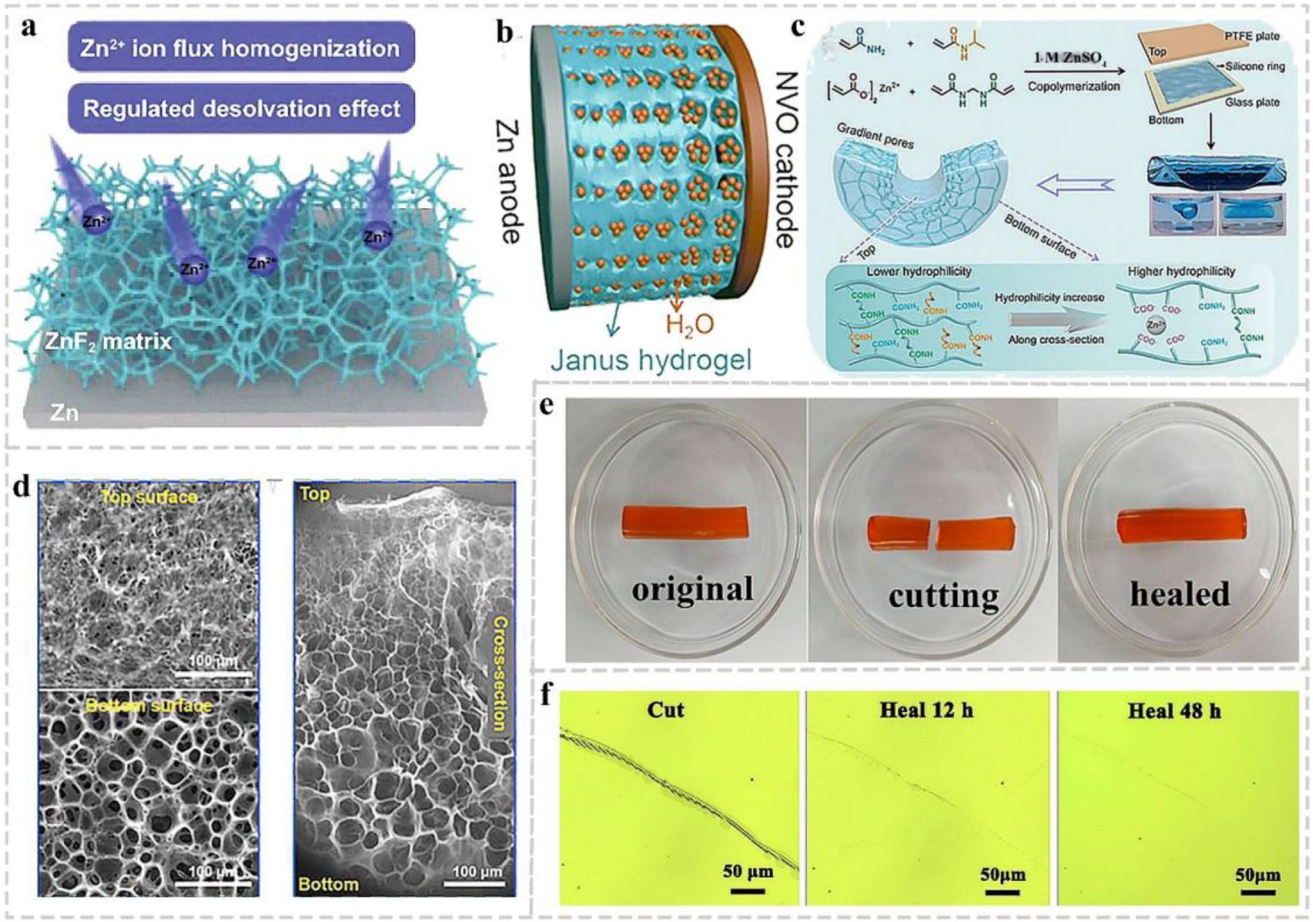
(a) Schematic illustration of the Zn@ZnF_2_ electrode. Reproduced under the terms of the Creative Commons CC BY license [80]. Copyright 2022. Springer Nature. (b) Zn||NVO full cell schematic with Janus PAZPM hydrogel electrolyte featuring gradient pores. (c) Preparation schematic, dyed photos, and 3D model of Janus hydrogel showing surface polymer composition. (d) Environmental scanning electron microscopy of Janus PAZPM hydrogel: surface views and cross‐sectional morphology. Reproduced with permission [198]. Copyright 2008, Royal Society of Chemistry. (e) the rod‐shaped specimens were cut into halves and self‐healed automatically into a complete hydrogel. (f) Microimages of the self‐healing process of the gels at distinct time intervals recorded by polarized optical microscopy. Reproduced with permission [85]. Copyright 2017, American Chemical Society.

In addition to enhancing the mechanical properties of the electrolyte bulk, constructing SEI with both stability and dynamic responsiveness is decisive for ensuring the long‐term cycling stability of the zinc anode [30, 194]. The zinc anode undergoes significant volume changes during charge/discharge cycles, causing traditional rigid SEI layers to experience periodic cracking and repair due to stress mismatch. This process not only accelerates the irreversible consumption of active materials and electrolytes but also continuously exposes fresh metal surfaces, providing active sites for the uncontrolled nucleation and growth of dendrites [118, 194, 195]. Consequently, developing smart interfacial layers capable of adapting to volume changes has become a key technological breakthrough. Current mainstream strategies focus on the structured design of artificial SEI layers, which can be achieved through in situ chemical reactions or ex situ coating processes [148, 196]. For example, introducing fluorine‐containing additives like fluoroethylene carbonate (FEC) into the electrolyte system, or directly depositing a composite inorganic layer such as ZnF_2_/ZnS on the zinc anode surface, can construct a dense interphase. This interphase combines high ionic conductivity (>10^−4^ S·cm^−1^) with electronic insulating properties (as shown in the interfacial morphology of the Zn@ZnF_2_ electrode in Figure 11a) [30, 31, 80]. Such interfaces regulate uniform Zn^2+^ deposition through a high interfacial energy effect while effectively suppressing parasitic side reactions like hydrogen evolution. However, a more innovative solution lies in constructing self‐adaptive interfacial systems utilizing dynamic covalent chemistry [30, 148, 197]. Among these, structurally designed adaptive interfaces have shown promise. A notable example is the Janus hydrogel electrolyte. This electrolyte features a continuous gradient in hydrophilicity/hydrophobicity and pore size along the thickness direction (as illustrated in Figure 11b–d). It maintains high water content on the cathode side to ensure capacity while creating a low water‐activity environment on the anode side. This design effectively suppresses side reactions such as corrosion and dendrite growth, demonstrating the adaptive regulation capability of gradient interfaces during volume changes [30]. Furthermore, researchers have leveraged dynamic covalent chemistry to elevate this adaptive capability to the molecular scale.

To validate the self‐healing properties of a dynamically cross‐linked gel, Shao et al. [85] conducted both macroscopic and microscopic tests on a PAA‐CNF‐Fe^3+^ hydrogel. As shown in Figure 11e, a rod‐shaped sample of the PAA‐CNF‐Fe^3+^ gel was cut with a blade. The two freshly cut surfaces were then brought into direct contact at room temperature (25°C) without applying external stress. The results show that the separated parts could autonomously reconnect and self‐heal, requiring no additional stimuli or healing agents. The healing kinetics were further elucidated through microscopic observation. Imaging with a polarized optical microscope at different time intervals (Figure 11f) revealed that surface cracks on the material essentially disappeared within 12 h and reached an almost completely healed state within 48 h. This result provides direct evidence of the highly efficient self‐healing capability of this physical gel. When the zinc anode undergoes volume contraction/expansion, this dynamic network enables the self‐healing of microcracks through molecular chain rearrangement. This process maintains tight mechanical coupling with the electrode surface, thereby eliminating issues of localized current overload and “dead zinc” formation [199, 200]. This dynamic adaptability not only ensures the physical integrity of the interface layer but also homogenizes deposition behavior by maintaining unimpeded ion transport channels. It fundamentally suppresses interface failure and uncontrolled dendrite growth caused by mechanical stress accumulation, offering a new material design paradigm for developing highly stable zinc‐metal batteries.

### 5.3 Optimize Solvation Structure to Suppress Side Reactions

As shown in the lower‐left quadrant of Figure 2, optimizing the solvation structure to suppress parasitic side reactions represents the other key design strategy. In FZABs, water molecules within the electrolyte are crucial for ensuring high ionic conductivity. However, their high electrochemical activity readily triggers a series of parasitic reactions at the zinc anode, such as hydrogen evolution and corrosion, and can also adversely affect the oxygen reaction kinetics at the air cathode [10, 12]. Therefore, a core objective for suppressing side reactions and enhancing overall cycling stability and energy efficiency lies in regulating the Zn^2+^ solvation structure and effectively inhibiting free water activity while maintaining good ionic conduction [10, 148]. Gel electrolytes, with their inherent three‐dimensional network structures, provide an ideal platform for achieving this balance. Through meticulous design of functional components and precise structural engineering, gel electrolytes enable the reconstruction of the electrode‐electrolyte interfacial environment at the molecular scale. This allows for the synergistic optimization of the reaction microenvironments at both the zinc anode and the air cathode, offering a critical pathway for developing high‐performance FZABs [62, 148].

**FIGURE 12:**
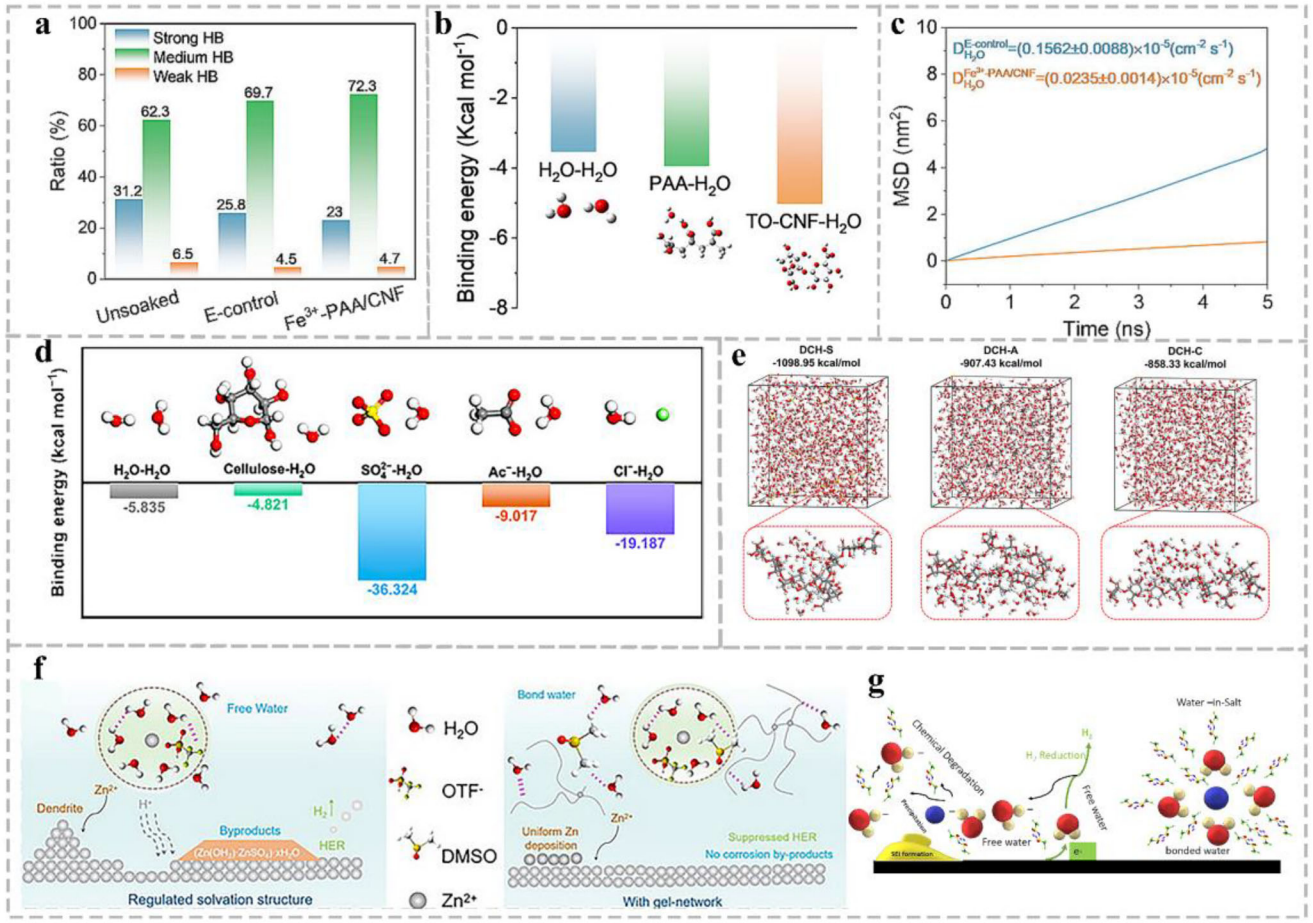
(a) The respective proportions of strong HB, medium HB, and weak HB in unsoaked, E‐control, and Fe^3+^‐PAA/CNF samples were calculated. (b) Binding energy of H_2_O‐H_2_O, PAA‐H_2_O, and CNF‐H_2_O obtained by DFT simulation. (c) Molecular diffusivity of water molecules in the Fe^3+^‐PAA/CNF hydrogel electrolyte and E‐control electrolyte. Reproduced with permission [65]. Copyright 2025, Wiley‐VCH GmbH. (d) The binding energy between H_2_O, SO_4_^2−^, Ac^−^, Cl^−^, and cellulose chains based on DFT calculations. (e) MD simulation snapshots and binding energy of DCH electrolytes. Reproduced with permission [144]. Copyright 2025, Wiley‐VCH GmbH. (f) Illustration of Zn^2+^ solvation and hydrogen bond changes in DN organohydrogel electrolyte. Reproduced with permission [204]. Copyright 2024, ELSEVIER B.V. and Science Press. All rights are reserved. (g) Illustration of the evolution of the metal ion solvation both water‐in‐salt and dilute solutions. Reproduced with permission [23]. Copyright 2020, Elsevier B.V. All rights reserved.

Employing a “water‐locking” strategy represents an effective method to directly reduce water activity. This approach involves introducing strongly hydrophilic substances into the gel matrix, which immobilize free water molecules through strong intermolecular interactions [26, 52, 201]. As shown in Figure 12a, the proportion of strong hydrogen bonds among water molecules significantly increases in the unsoaked sample compared to the E‐control sample. This is attributed to the monodentate coordination of OAc^−^ with K^+^, which releases carbonyl groups and enhances their interaction with water. Conversely, in the Fe^3+^‐PAA/CNF sample, the proportion of strong water hydrogen bonds decreases, due to the further strong binding of carboxyl groups in the network with water molecules. DFT calculations (Figure 12b) reveal that the binding energies for CNF‐H_2_O (−5.03 kcal·mol^−1^) and PAA‐H_2_O (−3.95 kcal·mol^−1^) are both lower than that for H_2_O‐H_2_O (−3.54 kcal·mol^−1^). This confirms that water molecules preferentially interact with CNF and PAA, disrupting the original hydrogen‐bond network among water molecules. Consequently, this suppresses water molecule activity and diffusion (Figure 12c), reduces side reactions, and improves battery performance. This mechanism also underpins the freeze‐resistant properties of this hydrogel electrolyte [65]. Zwitterionic polymers, such as poly (sulfobetaine methacrylate) (SBMA), which contain both positive and negative charge centers, exhibit a pronounced “anti‐polyelectrolyte effect” and exceptional hydration capability. When incorporated into a gel network, the sulfonate and quaternary ammonium groups on SBMA can capture numerous water molecules via ion‐dipole interactions, forming a tightly bound water layer [30, 86]. This significantly lowers the overall water activity of the system and effectively suppresses HER. To understand the mechanism of water immobilization by such polar/ionic groups at a microscopic level, methods commonly used in solvation structure studies can be referenced. For instance, DFT calculations and MD simulations can quantitatively evaluate the interaction strength between different components and water molecules [93, 202]. Yang et al. [144] on a cellulose‐based electrolyte system (Figure 12d) demonstrated that the binding energy for anion‐water pairs (e.g., SO_4_^2−^—H_2_O) is much lower than that for H_2_O—H_2_O, proving that anions can disrupt the original water network through strong hydration. MD simulations (Figure 12e) visually illustrate this process of anion insertion and reorganization of the hydration environment. Although the system studied is different, the core physical mechanism revealed—“strongly hydrating groups compete for and immobilize water molecules”—is consistent with the principle of SBMA reducing water activity via its zwitterionic groups. By analogy, the strongly polar sulfonate and quaternary ammonium groups in SBMA are expected to have binding energies with water molecules far exceeding those between water molecules themselves. This would firmly anchor water molecules to the polymer backbone, inhibiting their diffusion and reactivity, and ultimately enhancing the electrochemical stability of the electrolyte. A similar effect can be achieved by adding high‐boiling‐point, strongly polar organic co‐solvents, such as DMSO or glycerol. These co‐solvent molecules can not only form hydrogen‐bond networks with water molecules, reducing water's propensity to escape, but can also partially replace water molecules in the primary solvation sheath of Zn^2+^, forming mixed solvation structures like [Zn(H_2_O)_x_(Solvent)_y_]^2+^ (as shown in Figure 12f) [30, 72, 203]. This reconstruction of the solvation sheath alters the desolvation process of zinc ions and weakens the reactivity of water molecules at the electrode surface [16]. Consequently, it synergistically suppresses side reactions such as hydrogen evolution and corrosion from both thermodynamic and kinetic perspectives.

Constructing “Water‐in‐Salt” or “Water‐in‐Gel” electrolytes represents another effective strategy for restructuring solvation environments [17, 20, 26]. In electrolyte systems with high salt concentrations (typically exceeding 20 M), the abundant water molecules are “competed for” by cations and anions to form their respective solvation sheaths. This leaves virtually no freely mobile water molecules in the system [35, 93]. Under such conditions, anions (e.g., bis(trifluoromethanesulfonyl)imide (TFSI^−^), perchlorate (ClO_4_^−^)) are forced into the inner solvation sheath of Zn^2+^, forming contact ion pairs (CIPs) or aggregates (AGGs). This process not only alters the composition of Zn^2+^’s primary solvation sheath but also builds up an anion‐rich interfacial layer [23, 79, 205]. As illustrated in Figure 12g, this schematic depicts the evolution of the solvation structure in a “Water‐in‐Salt” electrolyte, including the distribution of free and bound water, the process of anions entering the Zn^2+^ solvation sheath, and the final formation of the anion‐enriched interphase [23]. This interphase layer can physically block the direct contact between the zinc anode and water molecules, significantly widening the electrochemical stability window, while concurrently suppressing HER. Integrating such high‐concentration electrolytes with a polymer network leads to the formation of “Water‐in‐Gel” electrolytes. This approach not only preserves the advantages of the “Water‐in‐Salt” system but also leverages the physical confinement effect of the gel scaffold to further immobilize solvent molecules. This enhances the suppression of side reactions and endows the electrolyte with excellent mechanical properties [30, 35, 93].

Centered on the core concept of solvation structure regulation and in response to the significantly different requirements of the cathode and anode in FZABs, the design philosophy of heterogeneous or functionally graded gel electrolytes has emerged. The Janus bilayer gel electrolyte is a prime example of this approach [43]. This design employs a bilayer architecture with asymmetric physical and chemical properties to achieve precise modification of the interfaces at both battery electrodes. The layer facing the zinc anode can be engineered to be hydrophobic or to homogenize ion flux. This interfacial layer works to suppress parasitic reactions such as hydrogen evolution, metal corrosion, and dendrite growth by simultaneously reducing local water activity and regulating Zn^2+^ flux. Conversely, the layer facing the air cathode is designed to be hydrophilic and rich in triple‐phase reaction interfaces. This facilitates efficient water management, ion transport, and oxygen diffusion, thereby promoting the kinetics of the ORR/OER [22, 30, 206, 207]. Similarly, constructing gel electrolytes with internal concentration or porosity gradients presents another ingenious strategy. For instance, establishing a gradient where salt concentration decreases from the anode side to the cathode side can create a high‐concentration, “Water‐in‐Salt”‐like environment near the anode to suppress side reactions, while maintaining suitably high ionic conductivity at the cathode to ensure unimpeded reaction rates [30, 35, 83]. Porosity gradient designs can simultaneously optimize dense deposition at the anode and efficient oxygen mass transport at the cathode [166, 208]. These innovative heterogeneous structural designs elevate electrolyte engineering from the modulation of solvation in homogeneous systems to the precise construction of the microenvironment at the electrode/electrolyte heterointerface. They offer a highly promising solution for tackling the long‐standing challenge of the conflicting reaction conditions at the cathode and anode in FZABs.

### 5.4 Innovative Structural Design for Ultra‐Thinness and Functional Integration

As presented in the lower‐right quadrant of Figure 2, innovating macroscopic architectures for ultrathin design and functional integration represents one of the core design strategies. Building upon the aforementioned strategies of regulating ion transport, enhancing mechanical stability, and optimizing the solvation microenvironment, the design of gel electrolytes is advancing toward the new frontier of macroscopic structural innovation and deep functional integration. This progression aims to meet the stringent requirements of flexible and wearable electronics for high energy density and integrated form factors. Two parallel and complementary technical pathways have emerged as critical: constructing gradient/heterogeneous structures and fabricating self‐standing ultra‐thin films. These innovative designs are not only focused on synergistically addressing the vastly different chemical environmental needs at the zinc anode and air cathode interfaces, but are also dedicated to breaking the physical thickness limitations of conventional batteries. This dual focus enables a significant increase in the overall cell energy density.

**FIGURE 13:**
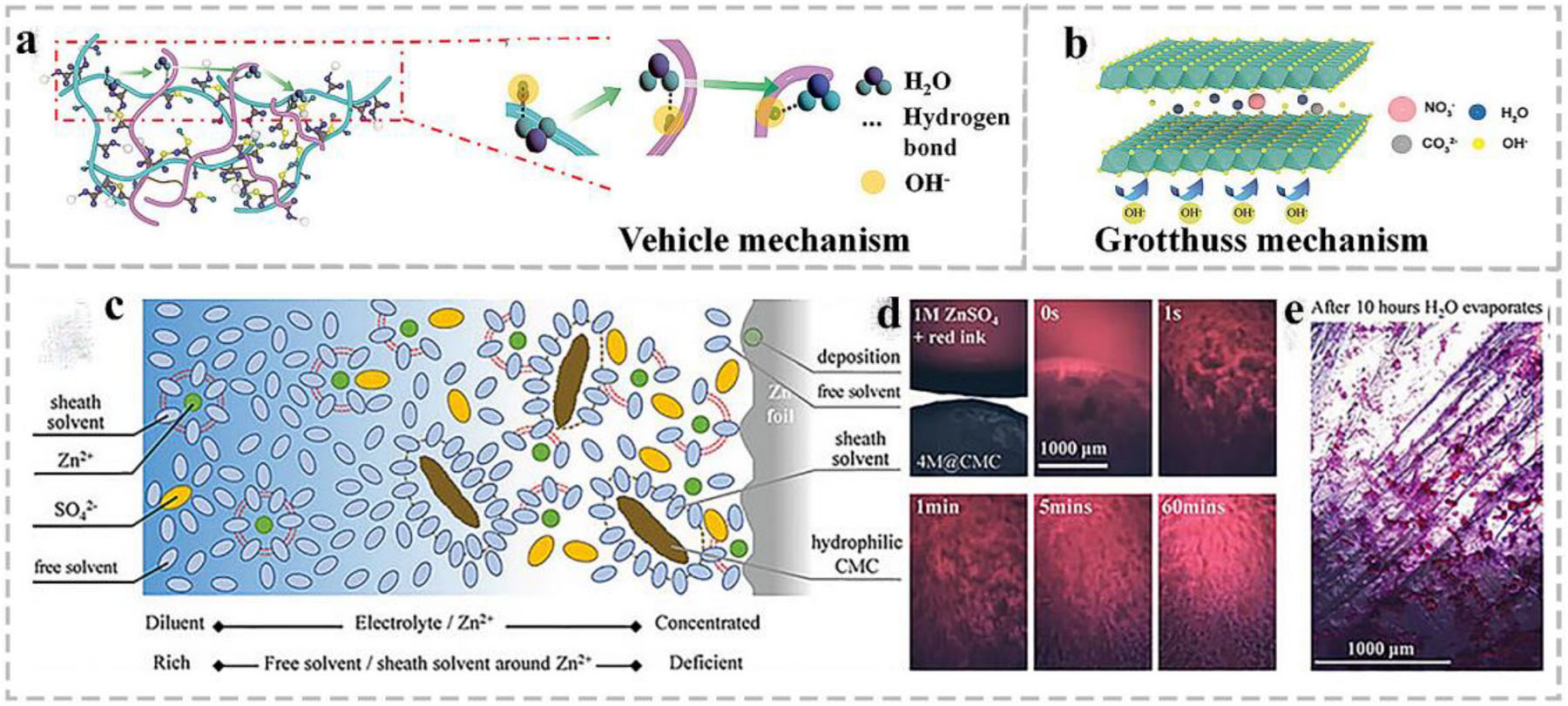
(a) Schematic diagram of the Vehicle transport mechanism of dual network hydrogels for OH^−^ and its localized magnification. (b) Schematic of the carrier pathway mechanism of Co/Al LDHs for OH^−^. Reproduced with permission [138]. Copyright 2024, Wiley‐VCH GmbH. (c) Solvation sheath schematic in a gradient electrolyte. (d) Gradient electrolyte formation snapshots. (e) Optical photo showing concentration gradient. Reproduced with permission [83]. Copyright 2023, Wiley‐VCH GmbH.

The design strategy of heterogeneous structures, particularly Janus bilayer gel electrolytes, provides an elegant approach for the precise regulation and separation of interfacial properties at the two electrodes of FZABs [36, 198]. As proposed by Yang et al. [138], a Janus strategy combines Grotthuss and Vehicle mechanisms by assembling a double‐network gel with layered double hydroxide (LDH) within the gel pores. The Co/Al‐LDH layer exhibits superior OH^−^ transport capability, with a significantly higher transport rate compared to the double‐network gel region. This difference can be explained by distinct proton conduction mechanisms: OH^−^ migration within the LDH primarily follows the Vehicle mechanism, whereas transport in the double‐network gel region aligns more closely with the Grotthuss mechanism (Figure 13a,b). The double‐network design of the gel phase substantially enhances the material's water absorption and retention, providing a hydrogen‐bond‐rich environment and carrier molecules for OH^−^. This promotes its transport via the Grotthuss mechanism—where OH^−^ forms [H_3_O_2_]^−^ intermediates with water molecules, reversibly decomposing into H_2_O and OH^−^ during movement. These two mechanisms coexist and act synergistically within the Co/Al‐LDH to ensure efficient ion migration [138]. Based on this, Janus electrolytes enable independent optimization of the cathode and anode interfacial environments by tailoring the composition and structure of their respective sides, thereby enhancing overall battery performance and stability. The core principle of this structure lies in constructing a dual‐faced electrolyte system with asymmetric physicochemical properties. The side facing the zinc anode (referred to as the zinc‐phobic layer) can be designed as a dense, hydrophobic structure or a polymer network rich in immobilized anionic groups [30, 36, 206]. Its functions are twofold: first, it effectively suppresses side reactions such as hydrogen evolution and metal corrosion by reducing local water activity; second, it forms a physical barrier through its high modulus and homogenizes ion flux, fundamentally inhibiting the nucleation and subsequent growth of zinc dendrites. Conversely, the side facing the air cathode (referred to as the oxygen‐philic layer) is designed to be hydrophilic and highly porous [206, 209, 210]. The design aims to maximize the triple‐phase reaction interface (the contact interface between the catalyst, electrolyte, and oxygen), ensuring efficient oxygen diffusion, adequate water supply, and rapid ion transport, thereby accelerating the sluggish kinetics of ORR/OER [30, 36, 210]. Similarly, gradient‐structured gel electrolytes with continuous property variations also demonstrate significant application potential. For instance, Luo et al. [30] summarized that introducing sodium CMC into the system can further regulate and maintain this beneficial concentration gradient. As shown in Figure 13c, the addition of CMC effectively reduces free water content and increases sulfate ion concentration, both of which contribute to thinning the Zn^2+^ solvation sheath. The resulting gradients in ion concentration, water content, and solvation structure from the anode outward directly influence zinc deposition behavior. Diffusion experiments using high‐ and low‐concentration ZnSO_4_@CMC solutions (Figure 13d,e) visually demonstrate the dynamic formation of this gradient within minutes and its continuous reinforcement upon evaporation. This provides an effective pathway for realizing an asymmetric electrolyte design that suppresses side reactions via high concentration at the anode interface while maintaining high ionic conductivity at the cathode side. This design philosophy, which regionalizes and integrates inherently conflicting functional requirements within a single electrolyte through a graded transition, achieves “compartmentalized regulation” of the internal battery microenvironment. It represents a key breakthrough for enhancing the comprehensive performance of FZABs.

Parallel to the functionalization of electrolyte structures, another significant trend is the push toward electrolyte ultrathinning. In flexible electronic devices, the electrolyte, as a non‐active component, directly influences the overall energy density and mechanical flexibility of the battery through its thickness [30, 206, 211]. Reducing the electrolyte thickness from the conventional scale of hundreds of micrometers down to the micrometer level is an extremely direct and effective method to enhance energy density [19, 211, 212]. Taking a composite hydrogel based on cellulose nanofibers (CNF) and graphene oxide (GO) as an example, this system leverages the excellent film‐forming ability and mechanical flexibility of cellulose as a supporting scaffold. Simultaneously, it incorporates the mechanical reinforcement and ion‐sieving functions provided by GO sheets. Even at an ultrathin scale, such an electrolyte can retain sufficient mechanical strength to effectively resist puncture by zinc dendrites [22, 213]. The successful fabrication of such ultra‐thin electrolytes significantly improves the energy density of full cells. Relevant studies report that reducing the electrolyte thickness from 200 µm to below 30 µm can increase the full‐cell energy density by approximately 40% [129]. These innovative structural designs not only propel zinc‐air batteries toward being lighter, thinner, and higher in energy density but also establish a broad developmental platform for future multifunctional integration, such as self‐healing and sensing capabilities.

## 6 Conclusions and Perspectives

To address the critical bottlenecks hindering the practical application of ZABs, such as uncontrolled zinc dendrite growth and electrode‐electrolyte interfacial instability and to meet the urgent demand for ultrathin, high‐energy power sources in flexible electronics, multi‐level cooperative design strategies based on gel polymer electrolytes have demonstrated significant technical advantages and achieved breakthrough progress. This review systematically summarizes the staged accomplishments attained through various approaches. These include regulating ion transport kinetics, enhancing mechanical buffering capacity, optimizing solvation coordination structures, and innovating macroscopic architecture design, and these strategies have collectively contributed to achieving uniform zinc anode deposition, physically blocking dendrite penetration, suppressing parasitic reactions, and improving the overall energy density of batteries. Current research indicates that functional gel electrolyte systems not only serve as an ideal replacement for conventional liquid electrolytes but also represent a core materials platform for advancing ZABs toward higher safety, longer cycle life, and superior mechanical flexibility.

**FIGURE 14:**
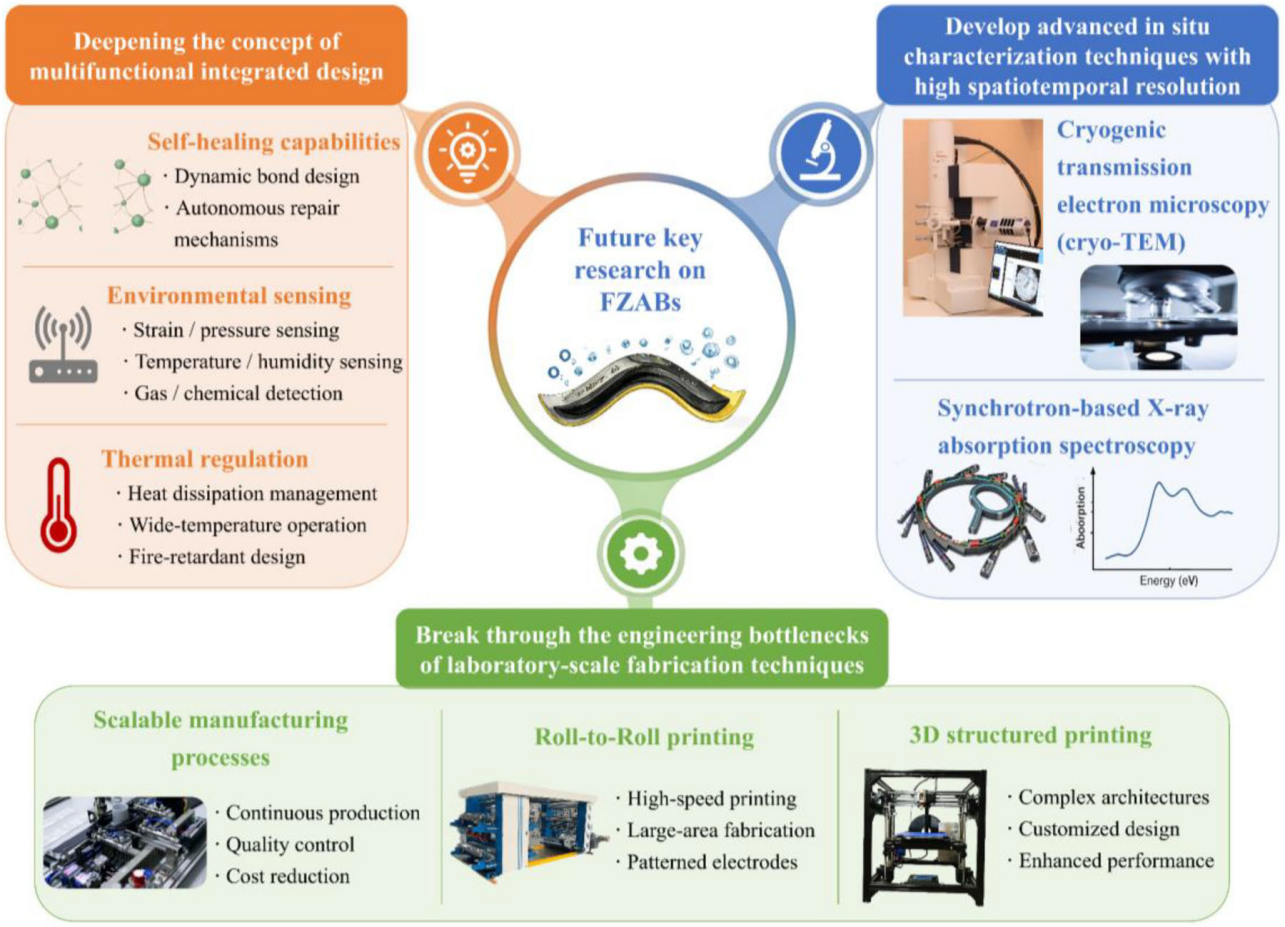
Diagram of future key research on FZABs.

Looking forward to the industrialization of FZABs, despite their promising technological prospects, several significant barriers must be overcome. To this end, future research must focus on three key directions (as shown in Figure 14):

First, deepening the concept of multifunctional integrated design is crucial. This involves the organic incorporation of intelligent modules‐such as self‐healing capabilities, environmental sensing, and thermal regulation‐into the electrolyte matrix. Specifically, self‐healing capabilities can be realized by introducing dynamic covalent bonds (e.g., boronate ester or disulfide bonds) or metal–ligand coordination (e.g., Fe^3+^‐carboxylate) into the polymer backbone, enabling autonomous repair of mechanical damage within minutes to hours under ambient conditions. For environmental sensing, conductive nanofillers such as carbon nanotubes or MXene can be integrated to construct strain‐ or temperature‐responsive conductive networks, allowing real‐time monitoring of battery deformation states via measurable resistance changes. Thermal regulation may be achieved by embedding phase‐change materials (e.g., paraffin@graphene microcapsules) within the gel matrix to buffer thermal runaway through latent heat absorption. The goal is to construct adaptive battery systems capable of actively responding to environmental changes.

Second, there is an urgent need to develop advanced in situ characterization techniques with high spatiotemporal resolution, such as cryogenic transmission electron microscopy (cryo‐TEM) and synchrotron‐based x‐ray absorption spectroscopy. Operationally, cryo‐TEM should be employed to quench and image the electrode‐electrolyte interface at specific cycling stages, resolving the nanoscale SEI morphology and crystallographic orientation of zinc deposits with sub‐nanometer resolution. Synchrotron x‐ray absorption near‐edge structure (XANES) and extended x‐ray absorption fine structure (EXAFS) spectroscopy, coupled with custom‐designed in situ electrochemical cells, can track the dynamic evolution of Zn^2+^ solvation sheaths and local coordination environments under realistic bending or stretching conditions. Additionally, operando Raman mapping combined with confocal microscopy offers a benchtop‐accessible approach to spatially monitor water activity gradients and ion concentration distributions across the electrolyte thickness during dynamic deformation. These techniques are essential for atomically resolving the structural evolution of the electrode‐electrolyte interface during dynamic deformation, thereby providing critical theoretical guidance for the rational design of materials.

Third, it is imperative to break through the engineering bottlenecks of laboratory‐scale fabrication techniques. The development of scalable manufacturing processes that combine cost‐effectiveness with precise control‐such as roll‐to‐roll printing and 3D structured printing‐is required [7]. For roll‐to‐roll processing, slot‐die coating of precursor solutions onto flexible substrates followed by UV‐initiated or thermal crosslinking enables continuous production of ultrathin gel electrolyte membranes with thickness uniformity controlled within ±5 µm at web speeds exceeding 10 m·min^−1^. For 3D structured printing, direct ink writing of shear‐thinning gel precursors containing programmable porogens allows layer‐by‐layer construction of gradient pore architectures with spatially varying crosslinking densities, achieving Janus‐type or hierarchical structures in a single manufacturing step. To ensure batch‐to‐batch consistency, in‐line rheological monitoring and machine‐learning‐assisted feedback control should be integrated to compensate for environmental humidity and temperature fluctuations during production. A primary focus must be solving engineering challenges related to the thickness uniformity, mechanical durability, and interfacial compatibility of ultra‐thin gel films. Through the synergistic advancement of fundamental scientific exploration, innovative material development, and engineering manufacturing technologies, there is substantial reason to believe that high‐performance FZABs based on advanced gel electrolyte systems will drive groundbreaking technological transformation in strategic fields such as wearable devices, internet of things terminals, and novel energy storage, thereby providing critical technological support for the development of intelligent and flexible energy systems of the future.

## Author Contributions

**Yapeng Cao**: wrote the manuscript; **Tengteng Gu**: and **Yuqiong Wen**: performed the formal analyses; **Xingxing Gao**:, **Jian Wang**:, and **Jun Liu**: revised the manuscript. All authors commented on the manuscript.

## Conflicts of Interest

The authors declare no conflicts of interest.

## Data Availability

The data that support the findings of this study are available from the corresponding author upon reasonable request.
